# An improved method for scoring protein-protein interactions using semantic similarity within the gene ontology

**DOI:** 10.1186/1471-2105-11-562

**Published:** 2010-11-15

**Authors:** Shobhit Jain, Gary D Bader

**Affiliations:** 1 Department of Computer Science, University of Toronto, 10 Kings College Road, Toronto, Ontario M5 S 3G4, Canada; 2 Banting and Best Department of Medical Research, Donnelly Centre for Cellular and Biomolecular Research, University of Toronto, 160 College St, Toronto, Ontario M5 S 3E1, Canada

## Abstract

**Background:**

Semantic similarity measures are useful to assess the physiological relevance of protein-protein interactions (PPIs). They quantify similarity between proteins based on their function using annotation systems like the Gene Ontology (GO). Proteins that interact in the cell are likely to be in similar locations or involved in similar biological processes compared to proteins that do not interact. Thus the more semantically similar the gene function annotations are among the interacting proteins, more likely the interaction is physiologically relevant. However, most semantic similarity measures used for PPI confidence assessment do not consider the unequal depth of term hierarchies in different classes of cellular location, molecular function, and biological process ontologies of GO and thus may over-or under-estimate similarity.

**Results:**

We describe an improved algorithm, Topological Clustering Semantic Similarity (TCSS), to compute semantic similarity between GO terms annotated to proteins in interaction datasets. Our algorithm, considers unequal depth of biological knowledge representation in different branches of the GO graph. The central idea is to divide the GO graph into sub-graphs and score PPIs higher if participating proteins belong to the same sub-graph as compared to if they belong to different sub-graphs.

**Conclusions:**

The TCSS algorithm performs better than other semantic similarity measurement techniques that we evaluated in terms of their performance on distinguishing true from false protein interactions, and correlation with gene expression and protein families. We show an average improvement of 4.6 times the *F*_1 _score over Resnik, the next best method, on our *Saccharomyces cerevisiae *PPI dataset and 2 times on our *Homo sapiens *PPI dataset using cellular component, biological process and molecular function GO annotations.

## Background

Gene Ontology (GO) [1] is a useful and popular taxonomy of controlled biological terms that can be used to assess the functional relationship between different gene products. GO organizes knowledge about gene function in a directed acyclic graph (DAG) of terms and their relationships. It is organized in three orthogonal ontologies capturing knowledge about cellular location, biological process and molecular function [1]. Experts annotate GO terms to genes in different organisms based on diverse evidence sources. GO has become the most used ontology and annotation system for assessing the confidence and biological relevance of high-throughput experiments based on the notion that if two or more genes are related by an experiment, they should also be related by known gene function. For instance, GO is often used as a benchmark for protein-protein interaction (PPI) experimental mapping and prediction [2-5], protein function prediction [6-8], and pathway analysis [9]. In this paper, we are specifically interested in the use of GO in a metric for protein-protein interactions (PPIs).

The relationship between gene products annotated to GO is quantified either simply from the annotated terms (for instance, by finding a set of common GO terms annotated to gene products) or more globally by using semantic similarity measures that consider the entire GO DAG. The GO DAG is a complex network of over 31,000 terms and 46,900 relations (GO release March, 2010). The cellular component ontology of GO describes gene product locations at the levels of sub-cellular structure and macromolecular complexes through over 2,650 terms and 5,000 relations. The molecular function ontology of GO is described using over 8,650 terms and 10,150 relations. The complexity of biological process ontology is even greater with over 18,500 terms and 38,700 relations. The large number of terms and relations describing the cellular knowledge covered by GO makes it difficult to naively quantify relationships between gene products. For example, *Saccharomyces cerevisiae *proteins Rpl10p (annotated to GO cellular component term 'large ribosomal sub-unit') and Sqt1p (annotated to GO cellular component term 'ribosome') physically interact with each other but do not share a GO term. Often, a sub-set of GO terms or a reduced version of GO, like *GO slim *[1], is used for relating genes. This makes GO terms and annotations easier to work with and compare, but valuable information is lost in the simplification.

Semantic similarity is a technique used to measure the likeness of concepts belonging to an ontology. Most early semantic similarity measures [10-12] were developed for linguistic studies in natural language processing. Recently, semantic similarity measurement methods have been applied to and further developed and tailored for biological uses [13-16]. A semantic similarity function returns a numerical value describing the closeness between two (or sometimes more) concepts or terms of a given ontology [17]. In the context of PPI datasets, semantic similarity can be used as an indicator for the plausibility of an interaction because proteins that interact in the cell (*in vivo*) are expected to participate in similar cellular locations and processes. For example, a high semantic similarity value between GO cellular component terms annotated to a set of proteins indicates that they are in close proximity and thus have a higher probability of interaction compared to proteins randomly selected from the proteome [2,4,18]. Thus, semantic similarity measures are useful for scoring the confidence of a predicted PPI using the full information stored in the ontology.

Semantic similarity measures can be broadly classified into two groups: edge based and node based. Edge based methods [19-22] determine semantic similarity based on the shared paths between two terms in a given ontology, whereas node based methods [10-12] rely on comparing the properties of the input terms (nodes), their ancestors, or descendants. One commonly used property is the specificity, or the information content (entropy), of the common ancestors between a pair of terms, which captures the notion of closeness in the DAG - the more specific the common ancestors of the terms, the closer the terms. The information content of a term *c *can be defined as the negative log likelihood of the term (eq. 1),

(1) IC(c)=−lnp(c)

where *p*(*c*) is the probability of occurrence of the term *c *in a specific corpus (e.g. GO annotations) [17]. While calculating *p*(*c*) in GO, the descendants of term *c *are also considered. For example, the probability of occurrence of the term 'cytosol' in the cellular component hierarchy of GO for *S*. *cerevisiae *defined by the number of genes assigned to it is 0.104 and its information content is 0.98. Comparative studies to determine the best semantic similarity measurement method have shown that performance on a variety of tests varies greatly depending upon the type of biological datasets used [23-26]. For example, for function prediction Resnik's [10] and Graph Information Content (simGIC) [25] work best and for cellular location prediction, the support vector machine (SVM) based method from Lei *et al*. [23] is preferable. In 2006, Guo *et al*. [24] compared a number of semantic similarity methods (Resnik [10] (AVG), Lin [11], Jiang [12], and graph similarity-based methods [27]) on a test to distinguish true from false human PPIs. They used proteins within a complex, or neighboring each other in Kyoto Encyclopedia of Genes and Genomes (KEGG) regulatory pathways, as a positive PPI dataset, and randomly chosen protein pairs as a negative interaction dataset. From receiver operating characteristic (ROC) curve performance analysis they concluded that Resnik (AVG) is better than other measures at distinguishing positive from negative PPIs. In 2008, Xu *et al*. [26] compared the Resnik [10] (MAX, AVG), Tao [15], Schlicker [13,28], and Wang [14] semantic similarity measurement methods on the same test with a *S. cerevisiae *PPI dataset from the Database of Interacting Proteins (DIP). They used Schlicker's rfunSimAll method which considers all GO ontologies together. From ROC analysis they found that the Resnik (MAX) method is best for GO ontologies taken separately or together. Thus, recent independent studies [24,26] show Resnik's method for calculating semantic similarity is best for measuring the likelihood of true PPIs.

Resnik's method defines semantic similarity between two ontology terms *s *and *t *for a given set *C *of common ancestors of *s *and *t *as,

(2) $$r(s,\;t)=\underset{c\in C}{\text{max}}[-\ln(p(c))]$$

where *p*(*c*) is the frequency of proteins annotated to term *c *and its descendants in the ontology. However, in most cases, proteins are assigned to more than one term in the same GO ontology. Suppose, proteins *A *and *B *are annotated to sets of GO terms *S *and *T*, respectively. The semantic similarity between *A *and *B *is defined as the maximum information content (Resnik (MAX)) of the set *S *× *T *(3).

(3) $$sim(A,\;B)=\underset{s_{i},t_{j},\in S,T}{\text{max}}r(s_{i},\;t_{j})$$

or as the average information content (Resnik (AVG)) of the set *S *× *T *(4).

(4) sim(A, B)=∑si,tj∈S,Tr(si,tj)n×m

where *s_i _*and *t*_j _are the GO terms in sets *S *and *T*, respectively, *r *(*s_i_, t_j_*) is the information content of the lowest common ancestor of terms *s_i _*and *t_j _*, and *n *and *m *are the set sizes. Resnik (MAX) has been found to be a better measure of likelihood for PPIs than AVG. The use of the MAX function with Resnik's method to score PPIs makes sense because proteins in a PPI only need to be in close proximity (similar cellular component terms) or in a similar biological process once, among all possible combinations annotation terms, for the PPI to be biologically relevant.

Resnik's measure calculates semantic similarity based only on the information content of a common ancestor. Therefore, it cannot differentiate between any two term pairs with same common ancestor even if they are in different parts of the GODAG. For example, proteins *A *and *B *annotated to the same cellular component term, e.g. 'cytoplasm', will have the same semantic similarity value as proteins *C *and *D *annotated to different terms, e.g. 'nucleus' and 'mitochondria', which have 'cytoplasm' as a common ancestor. Thus, Resnik's measure does not consider some of the information contained in the taxonomy by focusing only on the information content of a single ancestor term [29]. Lin's and Jiang's measures consider the information content of two terms along with that of a common ancestor but tend to overestimate similarity if the terms are higher up in the ontology [29]. For example, Lin's method will assign a score of 1 if two proteins are present in a same general compartment, e.g. 'cytoplasm'. Similar arguments also hold for molecular function and biological process GO ontologies. Further, the structure of GO is unbalanced with some paths having more details (depth) than others. This could be due to a particular path describing a more complex biological structure or to a particular focus of GO curators as they work to complete the ontology. For example, the 'intracellular' term of GO component has more depth than the 'extracellular' term (for *S. cerevisiae *GO DAG 'extracellular' term has a depth of 0 and 'intracellular' has a depth of 7), because there are many more biological terms associated with cell internals versus immediate cell externals. The ideal solution to these problems is to use a balanced GO DAG and annotation, but this is difficult to construct automatically [30]. Alternatively, we can develop semantic similarity scoring methods that consider the unbalanced natureof GO. In this paper, we have used the successful idea of information content from Resnik (MAX) and introduced clustering of similar GO terms into sub-graphs to create a new semantic similarity algorithm, Topological Clustering Semantic Similarity (TCSS), which outperforms Resnik's method for distinguishing positive from negative protein interactions and in other performance measurements.

## Results

### Algorithm

The goal of TCSS is to find subsets of GO terms defining similar concepts (e.g. nucleus related terms vs. mitochondrion related terms) and score gene products belonging to a similar subset higher than if they belong to different sets. In an effort to normalize the depth of the GO DAG across the ontology, the algorithm first defines mutually exclusive (non-overlapping) sub-graphs (sets of connected GO terms) rooted at major nodes. These sub-graphs are collapsed as single nodes to form a meta-graph and a two-level semantic similarity calculation is per-formed, as described below.

#### Topology based clustering

To normalize the depth of terms across the GO DAG, semantic similarity between terms, *s *and *t*, is calculated within a sub-graph instead of the complete GO graph. Sub-graphs consist of terms defining related concepts (e.g. all terms relating to the 'nucleus') and are defined based on a threshold on the information content of all terms present in a given ontology. The topological information content (ICT) of a term depends upon its specificity in the graph and is defined as shown in equation (5)

(5) $$ICT(t)=-\ln\left(\frac{\left|N(t)\right|}{\left|O\right|}\right)$$

where *t *is a term in the ontology *O*, |*N*(*t*)| is the number of child terms of *t*, and |*O*| is the total number of terms in *O *[31]. The terms which are more specific (i.e. terms which are present in the lower levels, closer to the leaves in the ontology graph) will have high information content as compared to less specific ones (i.e. terms which are present in the upper levels of the ontology graph closer to the root). An ICT cutoff (referred to as the 'topology cutoff')is defined in a pre-calculation step (see Methods) and terms below the cutoff are selected as sub-graph roots. If two sub-graph roots have similar ICT values (in the range of ± 20%) then their sub-graphs are merged. This is done to increase the dissimilarity between sub-graphs. A sub-graph contains all children of the selected sub-graph root term.

GO terms often have multiple parents, which could result in overlapping sub-graphs (a term is present in two sub-graphs). Each GO term in the cellular component ontology has on average 1.9 edges, whereas the ratio is 2.1 for the biological process ontology, and 1.2 for the molecular function ontology. All relationship types (or edges) are treated equally. Sub-graph overlap is removed in two steps (see Additional file 1: Supp. figure S1):

• *Edge removal by transitive reduction*. The GO DAG gives rise to partial orders ≤ on its vertices, where *u *≤ *v *when there exists a directed edge from *u *to *v*. However, *u *and *v *could connect via many different GO DAG paths. For example, the GO graph with paths *a *→ *b *→ *c *and *a *→ *c *has the same reachability as the GO graph with relation-ships *a *→ *b *→ *c*. Thus, the transitive reduction of GO graph *G *results in the smallest graph *R*(*G*) such that, the transitive closure of *G *is same as the transitive closure of *R*(*G*). This results in 14% and 6% fewer edges in cellular component and biological process ontologies respectively, reducing the likelihood of sub-graph overlap. There was no significant reduction in the molecular function ontology.

• *Term duplication*. After the reduction step, if a term still belongs to more than one sub-graph then it and its descendants are replicated in each sub-graph. Such a situation arises with a term having disjunctive ancestors (having independent paths from the ancestors to the term) belonging to different sub-graphs [32].

**Figure 1:**
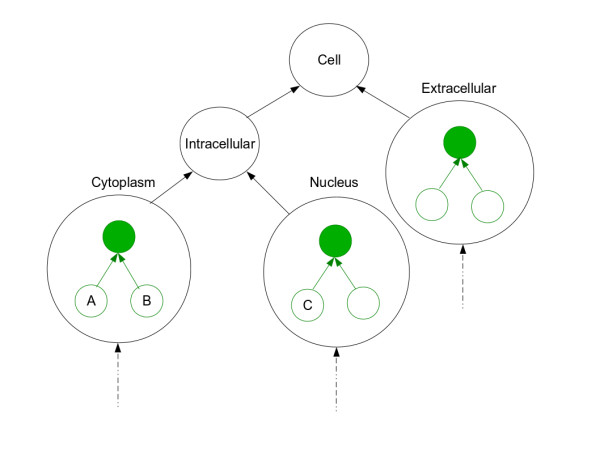
**Graphical illustration of the algorithm**. Nodes in the higher level graph and sub-graphs are shown by black and green circles, respectively. Root nodes of sub-graphs are shown by solid green circles and are equivalent to the corresponding higher level node. Terms A and B belong to the same sub-graph, therefore the semantic similarity score between them will be computed based on their common ancestor term 'Cytoplasm' (solid green). Terms B and C belong to different sub-graphs, therefore their semantic similarity score will be computed based on the common ancestor term 'Intracellular'.

Finally, all sub-graphs are connected into a hierarchy based upon the position of their root terms in original graph to construct a meta-graph (Figure 1). Meta nodes representing sub-graphs are labeled using the GO term of their sub-graph root.

#### Normalized scoring

We developed a system to calculate a semantic similarity score on the constructed meta-graph that results in more balanced semantic similarity scores compared to scoring the GO DAG directly. The system scores protein pairs in the same sub-graph higher than if they belong to different sub-graphs. The annotation information content (ICA) of all the terms present in an ontology is calculated based on the frequency of gene products annotated to a term and its children is shown in equation (6).

(6) $$ICA(t)\;=\;-\ln\left(\frac{annot(t)}{\sum_{t\in O}annot(t)}\right)\;$$

(7) $$annot(t)=|P_{t}\underset{c\in N(t)}{\cup }P_{c}|$$

where *t *is a term in the ontology *O *and *P_t _*is the set of gene products annotated to *t*. *N*(*t*) is the set of child terms of *t *in the ontology. The annotation information content values lie in the range [0, ∞) and are normalized to [0, 1] by dividing with the maxi-mum information content in a sub-graph or meta-graph. For a term $t_{i}^{s}$ belonging to the *i^th ^*sub-graph $G_{i}^{s}$, the sub-graph information content (ICS) of *t_i _*is defined as shown in equation (8).

(8) $$ICS(t_{i}^{s})=\frac{ICA(t_{i}^{s})}{\underset{{}_{t_{i}^{\text{s}}\in G_{i}^{\text{S}}}}{\max}ICA(t_{i}^{s})}$$

For a term $t_{i}^{m}$ in meta-graph *G^m^*, the information content (ICM) of *t_i _*is calculated as shown in equation (9).

(9) $$ICM\;(t_{i}^{m})=\frac{ICA(t_{i}^{m})}{\underset{{}_{t_{i}^{m}\in G^{m}}}{\max}ICA(t_{i}^{m})}$$

Gene products *A *and *B *may be annotated to more than one GO term. Let, *S *and *T *be the sets of GO terms annotated to gene products *A *and *B *respectively. Then the semantic similarity between gene products *A *and *B *is defined by the maximum approach, as shown in equation (10).

(10) $$\begin{array}{r} \underset{{}_{s_{\iota },t_{j}\in S,T}}{\max}\{\begin{array}{cc} ICM_{\max}(LCA(s_{i},t_{j})) & \text{if (a)}\\ ICS_{\max}(LCA(s_{i},t_{j})) & \text{if (b)} \end{array}\\ \begin{array}{rr} & (\text{a})\;s_{i}\in G_{i}^{s}\;\text{and }t_{j}\in G_{j}^{s}\\ & (\text{b})\;s_{i},t_{j}\in G_{i}^{s} \end{array} \end{array}$$

where *LCA*(*s_i,_t_j_*) is the lowest common ancestor (or the common ancestor with maximum information content) of the terms *s_i _*and *t_j _*. If both the terms *s_i_* and *t*_j_ belong to the same sub-graph then their lowest common ancestor will be in that sub-graph, otherwise it will belong to the meta-graph.

### Testing

In the previous section, we presented a new algorithm, Topological Clustering Semantic Similarity (TCSS), to compute semantic similarity between GO terms annotated to proteins that normalizes GO DAG branch depth. We compared the performance of TCSS with other semantic similarity measures given by Resnik [10], Lin [11], Wang *et al*. (Wang) [14], Schlicker *et al*. (simRel method) (Schlicker) [13], Jiang & Conrath (Jiang) [12], Pesquita *et al*. (SimGIC) [16] on the problem of scoring PPIs. Performance analysis of TCSS was done using receiver operating characteristic (ROC) and *F*_1_ measures. ROC grades the performance of classifiers as a trade-off between true positive rate (TPR) and false positive rate (FPR). We also used the *F*_1_ measure, which is the harmonic mean of precision (the proportion of retrieved information that is actually relevant) and recall (the proportion of relevant information that is retrieved) and indicates the classifier's ability to retrieve relevant information. The evaluation was done separately for cellular component (CC), biological process (BP), and molecular function (MF) ontologies.

#### Saccharomyces cerevisiae PPI test

*S. cerevisiae *positive and negative protein interaction sets (see Methods) were used to evaluate the above mentioned semantic similarity measures for their ability to distinguish positives from negatives. TCSS, Resnik, Lin, Jiang and Schlicker were tested using both the maximum (MAX) and best-match average (BMA) (see Methods) approach of combining multiple GO gene annotations and Wang was tested using only the BMA approach, as only BMA was used in the original Wang publication and is the only option available in the author's implementation. BMA averages scores when multiple combinations of GO terms are possible (for gene products annotated with multiple terms). SimGIC considers multiple GO annotations while calculating semantic similarity scores, thus MAX and BMA approaches are not relevant for it. We focused initial tests on manually annotated GO annotations ("without" annotations with IEA evidence codes (IEA-)), but also tested with all annotations, including electronic annotations ("with" annotations with IEA evidence codes (IEA+)).

**Figure 2:**
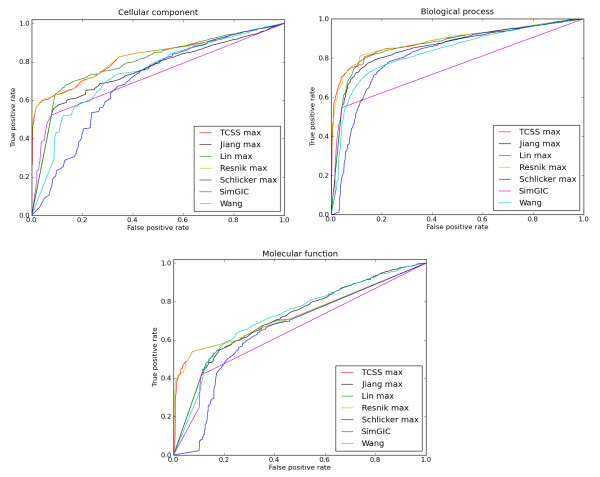
**ROC curves for *S. cerevisiae *PPI dataset**. ROC evaluations of semantic similarity measures at different cutoffs based on the *S*. *cerevisiae *PPI dataset derived from DIP are shown. The evaluation was performed using the cellular component, biological process and molecular function ontologies of GO. The maximum (MAX) approach for combining multiple annotations was used on the dataset, without (IEA-) electronic annotations. TCSS and Resnik show the best ROC profiles for all three ontologies.

**Figure 3:**
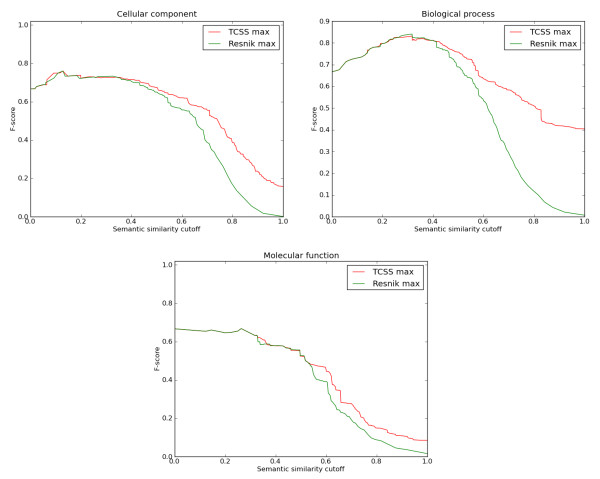
**F-score curves for *S. cerevisiae *PPI dataset**. F_1 _score (harmonic mean of precision and recall) evaluations of semantic similarity measures at different cutoffs based on the *S. cerevisiae *PPI dataset derived from DIP are shown. The evaluation was performed using cellular component, biological process, and molecular function ontologies of GO. Maximum (MAX) approach for combining multiple annotations was used on a dataset with only manual annotations (no electronic annotations (IEA-)). F_1 _score reaches its best value at 1 and worst at 0. TCSS does better than Resnik for semantic similarity cutoff scores in all three ontologies.

TCSS and Resnik consistently showed the best performance for all three ontologies in ROC analysis under different conditions (Table 1, Figure 2 (MAX, IEA-), Additional file 1: Supp. Figure S2 (BMA, IEA-), S4 (MAX, BMA, IEA+)). Since it is not clear from ROC analysis which of TCSS and Resnik performs better, we compared their *F*_1_ scores at different semantic similarity cutoffs for all the three ontologies (Figure 3 (MAX, IEA-), Additional file 1: Supp. Figure S3 (BMA, IEA+), S5 (MAX, BMA, IEA+)). TCSS showed average improvements of 6 times for CC, 5.9 times for BP, and 1.9 times for MF in retrieving relevant information over Resnik (Table 2) mainly due to the faster increase in true positive rate for TCSS at a given score threshold.

**Table 1:** Area under ROC curves for the *S. cerevisiae *PPI dataset

		IEA -			IEA+		
		CC	BP	MF	CC	BP	MF
TCSS	max	**0.83**	**0.89**	**0.73**	**0.83**	**0.89**	**0.75**
	bma	0.82	0.88	0.72	**0.83**	0.88	0.74

Resnik	max	**0.83**	**0.89**	**0.73**	**0.83**	**0.89**	**0.75**
	bma	0.81	0.87	0.72	**0.83**	0.88	0.74

Lin	max	0.80	0.87	0.70	0.79	0.87	0.72
	bma	0.79	0.85	0.68	0.80	0.86	0.72

Jiang	max	0.75	0.85	0.72	0.73	0.85	0.73
	bma	0.73	0.84	0.70	0.72	0.84	0.73

Schlicker	max	0.70	0.81	0.65	0.70	0.81	0.67
	bma	0.69	0.82	0.64	0.71	0.82	0.68

SimGIC		0.73	0.75	0.64	0.73	0.76	0.68

Wang		0.74	0.83	0.72	0.76	0.82	0.73

Tests were performed separately for cellular component (CC), biological process (BP) and molecular function (MF) ontologies. *Best-match average *and *maximum *approaches were used for datasets with (IEA+) and without (IEA-) electronic annotations. The best ROC scores are in bold.

**Table 2:** Improvement in F_1 _score for the *S. cerevisiae *PPI dataset

Best-match average						Maximun					
	IEA-			IEA+			IEA-			IEA+	
CC	BP	MF	CC	BP	MF	CC	BP	MF	CC	BP	MF
7.36	6.66	1.36	3.0	6.0	2.66	8.53	5.54	1.53	5.74	5.51	1.83

Average improvement in *F*_1 _scores achieved by TCSS over Resnik for best-match average and maximum approaches. TCSS does 6 times better than Resnik for cellular component (CC), 5.9 times for biological process (BP), and 1.9 times for molecular function (MF) ontologies, on average.

#### Homo sapiens PPI test

To test the generality of the method for PPI scoring, we ran similar tests as above using a H. *sapiens *PPI data set. H. *sapiens *positive and negative protein interaction sets (see Methods) were used to evaluate TCSS, Resnik, Lin, Jiang, Schlicker and SimGIC methods. The evaluation was done using BMA and MAX approaches for combining multiple GO annotations on IEA+/-datasets (Additional file 1: Supp. figures S6-S9, Supp. table S1). Table 3 shows the improvement in *F*_1_ scores achieved by TCSS over Resnik. On average TCSS performed 2.2 times better than Resnik for CC, 1.5 times for BP, and 2.5 times for MF ontologies.

**Table 3:** Improvement in F_1 _score for *H. sapiens *PPI dataset

Best-match average						Maximun					
	IEA-			IEA+			IEA-			IEA+	
CC	BP	MF	CC	BP	MF	CC	BP	MF	CC	BP	MF
3.44	1.51	2.42	1.28	1.64	4.0	2.7	1.48	2.0	1.53	1.58	1.50

Average improvement in *F*_1 _scores achieved by TCSS over Resnik for best-match average and maximum approaches. TCSS does 2.2 times better than Resnik for cellular component (CC), 1.5 times for biological process (BP), and 2.5 times for molecular function (MF) ontologies on average.

#### Correlation with gene expression

**Figure 4:**
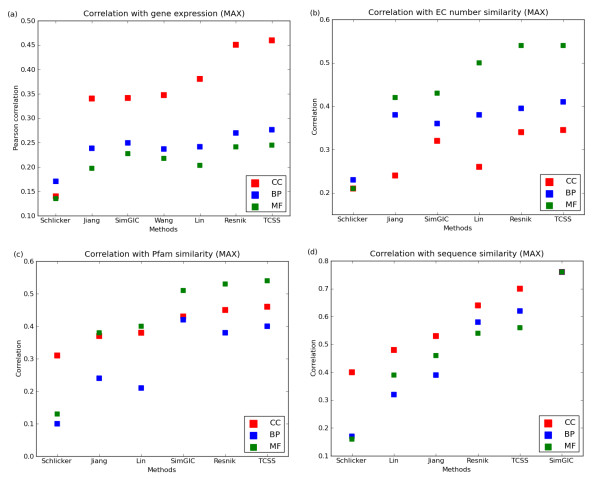
**Correlation with gene expression and CESSM dataset**. (a) Pearson correlation between gene expression similarity and semantic similarity on a *S*. *cerevisiae *dataset containing 5,000 randomly selected protein pairs are shown. (b - d) Correlation between semantic similarity and sequence, enzyme commission (EC), protein family (Pfam) similarity using the online CESSM tool. The evaluation was performed for cellular component (CC), biological process (BP), and molecular function (MF) ontologies of GO using maximum (MAX) approach for combining multiple GO annotations.

To test how our method performs in another application scenario, we tested its correlation with gene expression data. Two gene products that have similar function are more likely to have similar expression profiles and be annotated to similar GO terms [29].Therefore, a comparison of the similarity between gene expression of two gene products with the semantic similarity scores obtained by different measures can be used as a performance test. Gene expression profiles of randomly selected *S. cerevisiae *gene pairs (see Methods) were evaluated against the above mentioned semantic similarity methods. The evaluation was performed as above using the BMA/MAX approaches of combining multiple GO annotations on IEA+ dataset. TCSS showed the best correlation between gene expression and semantic similarity with all three GO ontologies (Figure 4(a), Additional file 1: Supp. Figure S16).

#### Correlation with EC, Pfam, and sequence similarity

The Collaborative Evaluation of GO-based Semantic Similarity Measures (CESSM) website was developed by Pesquita *et al*. [33] to evaluate semantic similarity measures on a standard set of data and benchmarks: correlation of similarity measure with similarity of sequence, Pfam domains and Enzyme Commission (EC) numbers (see Methods). We compared TCSS against Resnik, Schlicker, Jiang, Lin and SimGIC using CESSM for both MAX and BMA approaches on IEA-dataset. TCSS showed the best (or one of the best) correlation with EC similarity for all three ontologies (Figure 4(b), Additional file 1: Supp. Figure S17). For Pfam similarity using the MAX approach, TCSS is best for CC and MF ontologies and SimGIC is best for the BP ontology (Figure 4(c)). SimGIC better correlates with sequence similarity than other methods in all three ontologies (Figure 4(d), Additional file 1: Supp. Figure S17).

## Discussion

We present a new algorithm (TCSS) for calculating semantic similarity and tested its performance against other methods. TCSS shows an average improvement of 4.6 times in *F*_1_ scores over Resnik, the next best method, on our *S. cerevisiae *PPI test and 2 times on our *H. sapiens *PPI test. This clearly indicates the advantage of using TCSS to retrieve positive protein interactions and hold back negative interactions over Resnik's method. We compared TCSS using both the BMA and MAX approaches for combining multiple GO annotations, and found that MAX generally works best for PPI datasets. The use of the MAX function to score PPIs, instead of an 'average' function, makes sense because proteins in PPIs only need to be in close proximity (similar cellular component terms) or in a similar biological process once, among all possible combinations annotation terms, to be biologically relevant. Therefore, the MAX approach is unlikely to overestimate true PPIs. However, there may be application scenarios (e.g to compute a more general measure of functional similarity) where the MAX approach could lead to over-estimation and BMA would be a better choice. In these cases, TCSS can be modified to use the BMA method instead of MAX (see Methods). For example, TCSS shows worse correlation with Pfam similarity than SimGIC on the biological process ontology test, but becomes better when using BMA (see Additional file 1: Supp. Figure S17). Also, it is evident from the correlation of semantic similarity with gene expression similarity that TCSS is more likely to assign a higher score to gene products if they also exhibit similar gene expression. Tests using the CESSM benchmark dataset were in favor of TCSS for EC number similarity and Pfam similarity. SimGIC does better than TCSS in the sequence similarity correlation test. One reason for this could be that SimGIC scores gene products with shared annotation terms and gene products annotated to same term are more likely to be part of the same gene family and thus have high sequence similarity.

**Figure 5:**
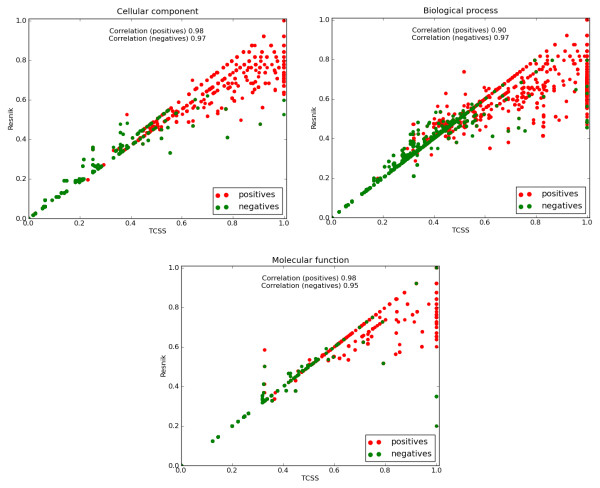
**Comparison of our topological clustering method and Resnik (MAX) as scoring positive and negative PPIs**. The scatter plot of semantic similarity scores for positive (red) and negative (green) interactions. Semantic similarity scores range between 0.0 and 1.0 for both methods, with 1.0 being the best. A significant number of positive interactions are under-scored by Resnik (MAX) in all three ontologies compared to TCSS.

Scatter plots of the semantic similarity scores obtained by TCSS (MAX) and Resnik (MAX) methods clearly indicate that a significant number of positive protein interactions are under-scored by Resnik (Figure 5) in all three ontologies (p-values by Kolmogorov-Smirnov test: Cellular component: 6.4e-59, Biological process: 3.4e-163, Molecular function: 1.6e-15). Given below are some biological examples selected from these scatter plots in support of our claim:

• **Cellular component: **Rpl10p is a *S. cerevisiae *protein responsible for joining of the 40 S and 60 S ribosomal subunits [34]. It has been found to interact [35-38] with Sqt1p, an essential protein involved in a late step of 60 S ribosomal subunit assembly or modification [34] using affinity capture-mass spectrometry (MS), affinity capture-western and two-hybrid experimental methods. Rpl10p is annotated to the 'cytosolic large ribosomal subunit' term and Sqt1p is annotated to the 'cytosolic ribo-some' term [1]. The score assigned by Resnik (MAX) to the Rpl10p-Sqt1p interaction is 0.4 which is low considering that both the proteins are in similar cellular components and the 'cytosolic large ribosomal subunit' term is the child term of 'cytosolic ribosome' in GO. The same interaction gets a score of 0.78 by TCSS (MAX), which categorizes it as a high confidence interaction, due to the normalization step on the 'cytosolic ribosome' sub-graph.

• **Biological process: **The Nth1p-Dcs1p protein-protein interaction was experimentally shown by Uetz *et al *(2000) and Yu *et al *(2008) [39,40] using two-hybrid experiments. Both Nth1p and Dcs1p proteins share the 'vacuolar protein catabolic process' term in GO [1]. The score assigned by Resnik (MAX) to the Nth1p-Dcs1p interaction is 0.45 which is low considering that both proteins are part of the same biological process. The same interaction gets a score of 1 by TCSS (MAX), due to the normalization on 'vacuolar protein catabolic process' sub-graph, thus categorizing it as a high confidence interaction.

• **Molecular function: **Mft1p and Hpr1p are the subunits of the nuclear THO complex, which is involved in transcription elongation, mitotic recombination and telomere maintenance [34]. The Mft1p-Hpr1p interaction has been shown by affinity capture-MS and affinity capture-western experimental techniques [35,41-43]. Both Mft1p and Hpr1p are an-notated to the 'nucleic acid binding' term of GO [1]. This interaction is assigned a score of 0.2 by Resnik (MAX) because the term nucleic acid binding is fairly general. This score is low considering that both the proteins are part of a same GO term. The same interaction is assigned a score of 1 by TCSS (MAX), due to the normalization step on the 'nucleic acid binding' sub-graph. 'Nucleic acid binding' is a general molecular function term with a shallow hierarchy.

Future directions for TCSS development include testing if the GO graph edge type (e.g. is-a, part-of) can provide additional information that will lead to improved performance and also testing the method more rigorously with other data sets.

## Conclusions

We present a new semantic similarity algorithm, Topological Clustering Semantic Similarity, designed to use the GO for PPI confidence assessment. It partitions the GO DAG into non-overlapping sub-graphs, using a topological clustering method, and computes semantic similarity normalized within each sub-graph. We evaluated TCSS against other methods for measuring semantic similarity between GO terms annotated to proteins involved in protein-protein interactions from *S. cerevisiae *and *H. sapiens*. We also tested the correlation between multiple semantic similarity scoring methods with gene expression, protein sequence, EC, and Pfam similarity. Performance tests were generally in favor of TCSS in all three GO ontologies: cellular component, biological process and molecular function.

This new method will be useful as an evidence source in PPI prediction or in confidence assessment of PPI datasets.

## Methods

### Data acquisition and processing

• **Ontology data: **Ontology data was down-loaded from the Gene Ontology database [1] (dated March 2010) containing 31,382 ontology terms subdivided into 2,689 cellular component, 18,545 biological process and 8,688 molecular function terms.

• **GO Annotation data: **Gene annotations for GO terms were downloaded from the Gene Ontology database for *S. cerevisiae *(dated February 2010) [44] and *H. Sapiens *(dated August 2010) [45]. Electronically inferred annotations (IEA) lack manual review therefore, we de-signed two sets of tests, one with IEA annotations and one without. In our implementation, we only consider the most specific GO gene an-notations. For example, if gene A is annotated to terms × and Y (and × is an ancestor of Y), then we only consider annotation to Y. This is because in ontologies a term is a aggregate of its descendants. This pre-filtering of GO could impact the results of some methods used in our analysis. For instance, in CESSM tests, correlation between SimGIC semantic similarity and EC similarity for the molecular function ontology increases by 25% and correlation with sequence similarity decreases by 15% if all the annotations are considered, however all other changes we noticed were minor and didn't change our results.

• **Interaction dataset: **To evaluate the performance of TCSS against other semantic similarity measures on the problem of scoring PPI confidence, we created positive and negative interaction datasets for *S. cerevisiae *and *H. sapiens*.

-*S. cerevisiae: *We retrieved 4,598 unique pairwise *S. cerevisiae *PPIs from the core set of Database of Interacting Proteins (DIP) (dated December 2009) [46]. The DIP core database records data derived from both small-scale and large-scale experiments that have been validated by the occurrence of the interaction between paralogous proteins in different species [47]. The positive dataset for CC, BP, and MF ontologies comprised interactions with both proteins annotated to terms (other than root) in their respective ontologies (see Table 4). The negative dataset with the same number of PPIs as the positive set was generated by randomly selecting proteins from genes in the GO annotation files that are not known to be positive in a set of all known (45,448) yeast PPIs from iRefWeb (September 2010), a meta-database containing the ten largest primary PPI databases [48].

**Table 4:** Distribution of positive and negative interactions

	*S. cerevisiae*		*H. sapiens*	
	IEA+	IEA-	IEA+	IEA-
CC	4469	4425	1431	1054
BP	4385	4326	1435	1204
MF	3858	3583	1441	1288

Number of interactions in the positive dataset for cellular component (CC), biological process (BP), and molecular function (MF) ontologies.

-*H. sapiens: *We retrieved 2; 077 unique pairwise PPIs (with three or more publications) for *H. sapiens *from DIP (dated June 2010). The positive dataset for CC, BP, and MF ontologies comprised interactions with both proteins annotated to terms (other than root) in their respective ontologies (see Table 4). The negative interaction dataset contained an equal number of randomly selected interactions from a pool of all possible interactions in human minus all known (43,935) iRefWeb [48] known PPIs.

• **Gene expression datasets: **The gene expression dataset for *S. cerevisiae *was down-loaded from GeneMANIA [49] (dated August 2010) and contained data from 39 different microarray experiments (see Additional file 1: Supp. table S2). Test datasets were prepared from 5; 000 *S. cerevisiae *gene pairs randomly selected from a list of all possible pairs of proteins in our gene expression data set, including an equal number of random and known PPIs (PPIs in the DIP core set have higher than average expression correlation). This was done independently for CC, BP, and MF annotations of GO (including IEA annotations).

• **CESSM dataset: **Collaborative Evaluation of GO-based Semantic Similarity Measures (CESSM) is an online tool for the automated evaluation of GO-based semantic similarity measures in terms of performance against sequence, Pfam (protein family) and EC (enzyme commission number) similarity [33]. Protein pair (from multiple species), GO (dated August 2010), and UniProt GO annotations (dated August 2008) were downloaded from CESSM.

### Algorithm implementation

The algorithm as described in the results section was implemented using the Python programming language [50]. An important step in our algorithm is to determine the size of sub-graphs. This is determined by thresholding the topological information content (ICT) of terms in a given ontology (see Results). The cutoff is chosen to maximize performance (AUC and *F*_1_measures) on a given benchmark/test. The relationship between AUC and topology cutoff follows a U - shaped curve with a global maximum for all three ontologies. Average F-score shows a general upward trend with topology cutoffs (see Additional file 1: Supp. figures S10, S11, S13, S14). A topology cutoff must be computed for each test before we compute semantic similarity scores, which is a practical disadvantage of our method, though we expect cutoffs to be useful generally for a type of data and an organism, once computed. Topology cutoffs for different datasets are as follows:

• *S. cerevisiae *PPI dataset: 2.4 for CC, 3.6 for BP, and 3.2 for MF (see Additional file 1: Supp. Figure S12)

• *H. sapiens *PPI dataset: 3.0 for CC, 4.0 for BP, and 3.6 for MF (see Additional file 1: Supp.Figure S15)

• Expression dataset: 2.4 for CC, 3.6 for BP, and 3.2 for MF

• CESSM dataset: 3.4 for CC, 3.2 for BP, and 3.0 for MF

Our results are resilient in the immediate cutoff range of ± 0.1 for all three ontologies.

#### Best-match average approach

Let, *S *and *T *be the sets of GO terms annotated to gene products *A *and *B *respectively. Then semantic similarity between gene products *A *and *B *based upon the best-match average approach [14,51] is defined by the equation (11).

(11) $$\begin{array}{l} \frac{\sum_{s_{i}\in S}Sim(s_{i},\;T)+\sum_{t_{j}\in N}Sim(t_{j},\;S)}{\left|S|+|T\right|} \end{array}$$

where *Sim *(*u_i_, V *) is defined as (12),

(12) $$\begin{array}{r} \underset{{}_{{}_{v_{j}\in V}}}{\max}\{\begin{array}{cc} ICM_{\max}(LCA(u_{i},v_{j})) & \text{if (a)}\\ ICS_{\max}(LCA(u_{i},v_{j})) & \text{if (b)} \end{array}\\ \begin{array}{rr} & (\text{a})\;u_{i}\in G_{i}^{s}\;\text{and }v_{j}\in G_{j}^{s}\\ & (\text{b})\;u,v_{j}\in G_{i}^{s} \end{array} \end{array}$$

### Algorithm analysis

#### Other methods

Semantic similarity measurement methods given by Resnik [10], Lin [11], Schlicker *et al*. (simRel method) (Schlicker) [13], Jiang & Conrath (Jiang) [12], and Pesquita *et al*. (SimGIC) [16] were implemented as described in their respective publications. The GOSemSim [52] implementation in R was used for Wang *et al*. (Wang) [14].

#### ROC and F-measure

Different measures used for analyzing the performance of our algorithm are as follows:

• True positive rate (TPR), also known as Recall:

(13) $$TPR\;=\frac{TP}{TP+FN}$$

• False positive rate (FPR):

(14) $$FPR\;=\frac{FP}{FP+TN}$$

• Precision (P):

(15) $$P=\frac{TP}{TP+FP}$$

• F_1_ measure (F):

(16) $$F=2\frac{P\times TPR}{P+TPR}\;\;$$

• Improvement in F_1 _score is calculated as the average improvement at different semantic similarity cutoffs.

• Area under curve (AUC) was calculated using the trapezoidal rule.

where *TP*, *FP*, *TN*, *FN *are true positive, false positive, true negative, and false negative, respectively.

#### Correlation with gene expression

Average gene expression Pearson correlation was calculated for the *S. cerevisiae *positive and negative interaction dataset using Fisher's *z *transformation [53].

(17) $$z_{n}=1/2\ln\left(\frac{1+r_{n}}{1-r_{n}}\right)$$

where *r_n _*is the Pearson correlation between two genes for the *n*^th ^experiment. Then, an appropriate estimate of the true mean is calculated as,

(18) $$\bar{z_{n}}=N^{-1}\sum_{i=1}^{N}z_{i}$$

where *N *is the total number of experiments. Then, by inversion, average correlation is calculated as,

(19) $$\bar{r_{n}}=\frac{e^{2\bar{z_{n}}}-1}{e^{2\bar{z_{n}}}+1}$$

#### CESSM evaluation

TCSS, Schlicker, Jiang, SimGIC and Resnik methods were used to find the semantic similarity between protein pairs provided by CESSM. Correlation between semantic similarity scores and sequence, Pfam, EC similarity for these methods was calculated using the CESSM online tool. Wang was not used here due to the difficulty in modifying the R implementation to use the datasets provided by the CESSM website.

### Availability and requirements

• Project name: Topological Clustering Semantic Similarity (TCSS)

• Home page: http://baderlab.org/Software/TCSS

• Operating system(s): Unix/Linux (recommended)

• Programming language: Python

• Other requirements: Python 2.6 or higher

• License: GNU LGPL

• Any restrictions to use by non-academics: no

## Authors' contributions

SJ conceived the study and carried out all programming and analysis as a Ph.D. student in the lab of GB. GB supervised and provided input on all aspects of the study. All authors read and approved the final manuscript.

## Supplementary Material

Additional file 1 **Supplementary figures and tables**. The file contains supplementary figures S1 - S17 and tables S1 & S2. Click here for file
