# Supplementary material for: An improved method for scoring protein-protein interactions using semantic similarity within the gene ontology
Source: BMC Bioinformatics. 2010 Nov 15;11:562. doi: 10.1186/1471-2105-11-562 (PMC2998529; doi:10.1186/1471-2105-11-562)
Supplement: Additional file 1 — Supplementary figures and tables. The file contains supplementary figures S1 - S17 and tables S1 & S2. [file 1471-2105-11-562-S1.PDF]

(Supplementary material)
An improved method for scoring protein-protein interactions using semantic similarity within the Gene Ontology

Shobhit Jain[1,2], Gary D. Bader*[1,2]

[1]Department of Computer Science, University of Toronto, 10 Kings College Road, Toronto, Ontario M5S 3G4, Canada
[2]Banting and Best Department of Medical Research, Donnelly Centre for Cellular and Biomolecular Research, University of Toronto, 160 College St, Toronto, Ontario M5S 3E1, Canada

Email: Shobhit Jain - shobhit@cs.toronto.edu; Gary D. Bader*- gary.bader@utoronto.ca;

*Corresponding author

Supplementary figure S1 - Mutually exclusive sub-graphs

(a) Transitive reduction - suppose a, b, c, and d are the nodes in graph G with directed edges as shown in figure (a). Let the number of genes annotated to each node is 1. Then the total annotation of node a in G (annotation of a and its descendants) is 4. Transitive reduction of G will result in G' without edge $d \rightarrow a$ and with same total annotation of 4 as a can still be reached from d. (b) Replication - suppose term d is common to both the sub-graphs B and C then term d will be copied to both the sub-graphs.

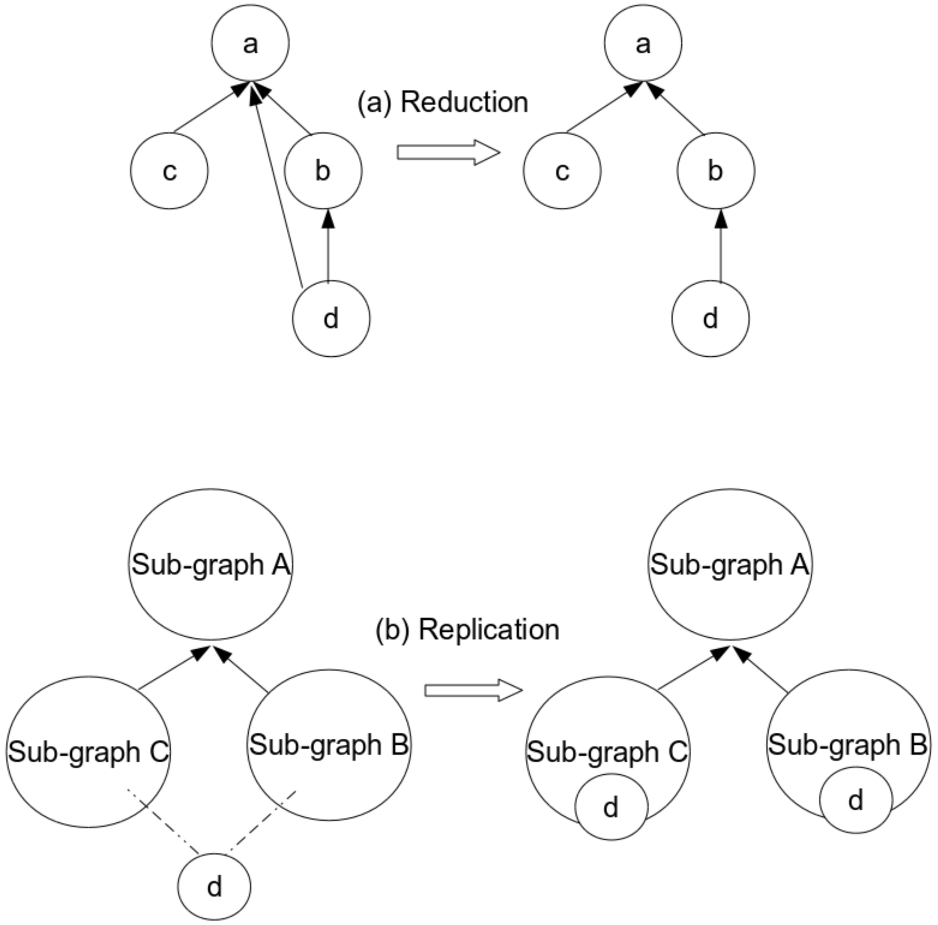

Supplementary figure S2 - ROC curves for *S. cerevisiae* PPI dataset (IEA-)

ROC evaluations of semantic similarity measures at different cutoffs based on the *S. cerevisiae* PPI dataset derived from DIP are shown. The evaluation was performed using cellular component, biological process, molecular function ontologies of GO. Best-match average (bma) approach for combining multiple annotations was used on dataset without (IEA–) electronic annotations. TCSS & Resnik show best ROC profiles for all three ontologies.

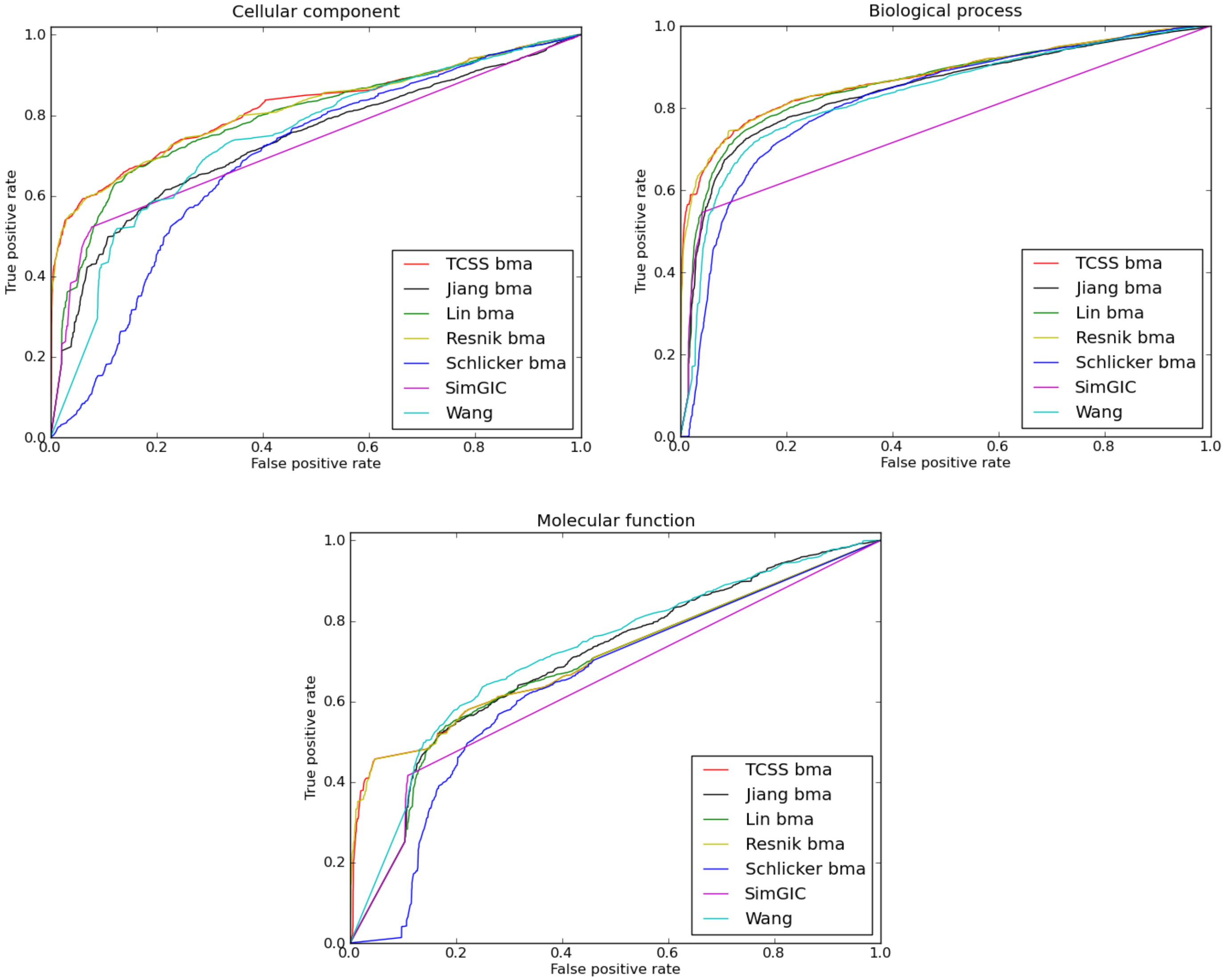

Supplementary figure S3 - F-score curves for *S. cerevisiae* PPI dataset (IEA-)

F_1 score (harmonic mean of precision and recall) evaluations of TCSS and Resnik semantic similarity measures at different cutoffs based on the *S. cerevisiae* PPI dataset derived from DIP are shown. The evaluation was performed using cellular component, biological process, molecular function ontology of GO. Best-match average (bma) approach for combining multiple annotations was used on dataset without (IEA–) electronic annotations. F_1 score reaches its best value at 1 and worst at 0. TCSS does better than Resnik for semantic similarity cutoff scores in all three ontologies.

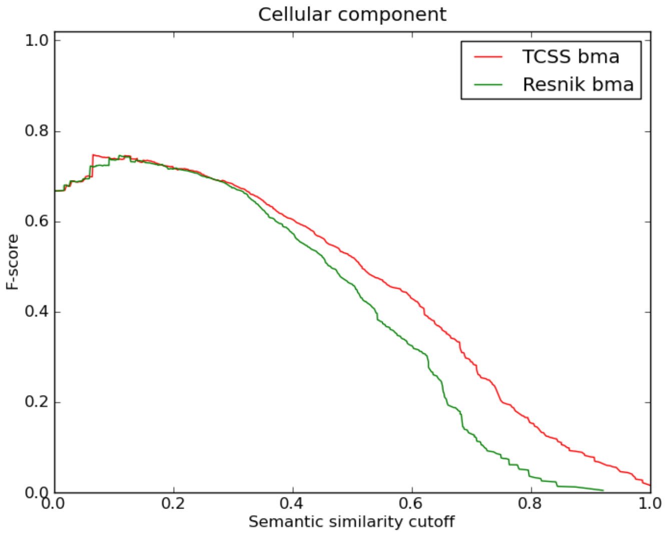

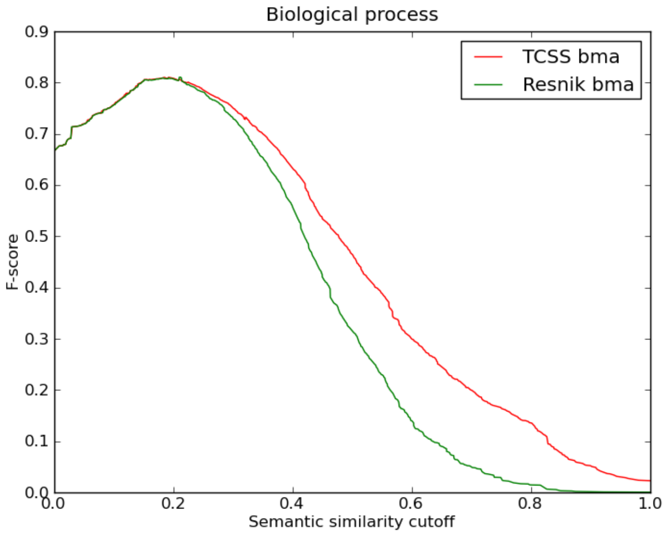

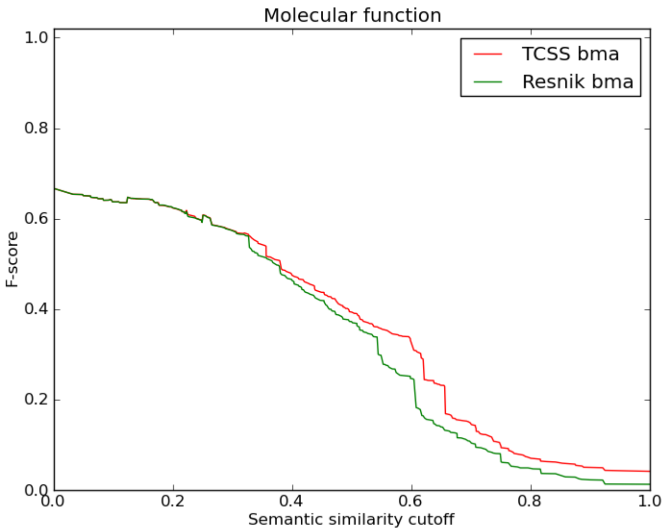

Supplementary figure S4 - ROC curves for *S. cerevisiae* PPI dataset (IEA+)

ROC evaluations of semantic similarity measures at different cutoffs based on the *S. cerevisiae* PPI dataset derived from DIP are shown. The evaluation was performed using cellular component, biological process, molecular function ontology of GO. Best-match average (bma) and maximum (max) approaches for combining multiple annotations are used on dataset with (IEA+) electronic annotations. TCSS & Resnik show best ROC profiles for all three ontologies.

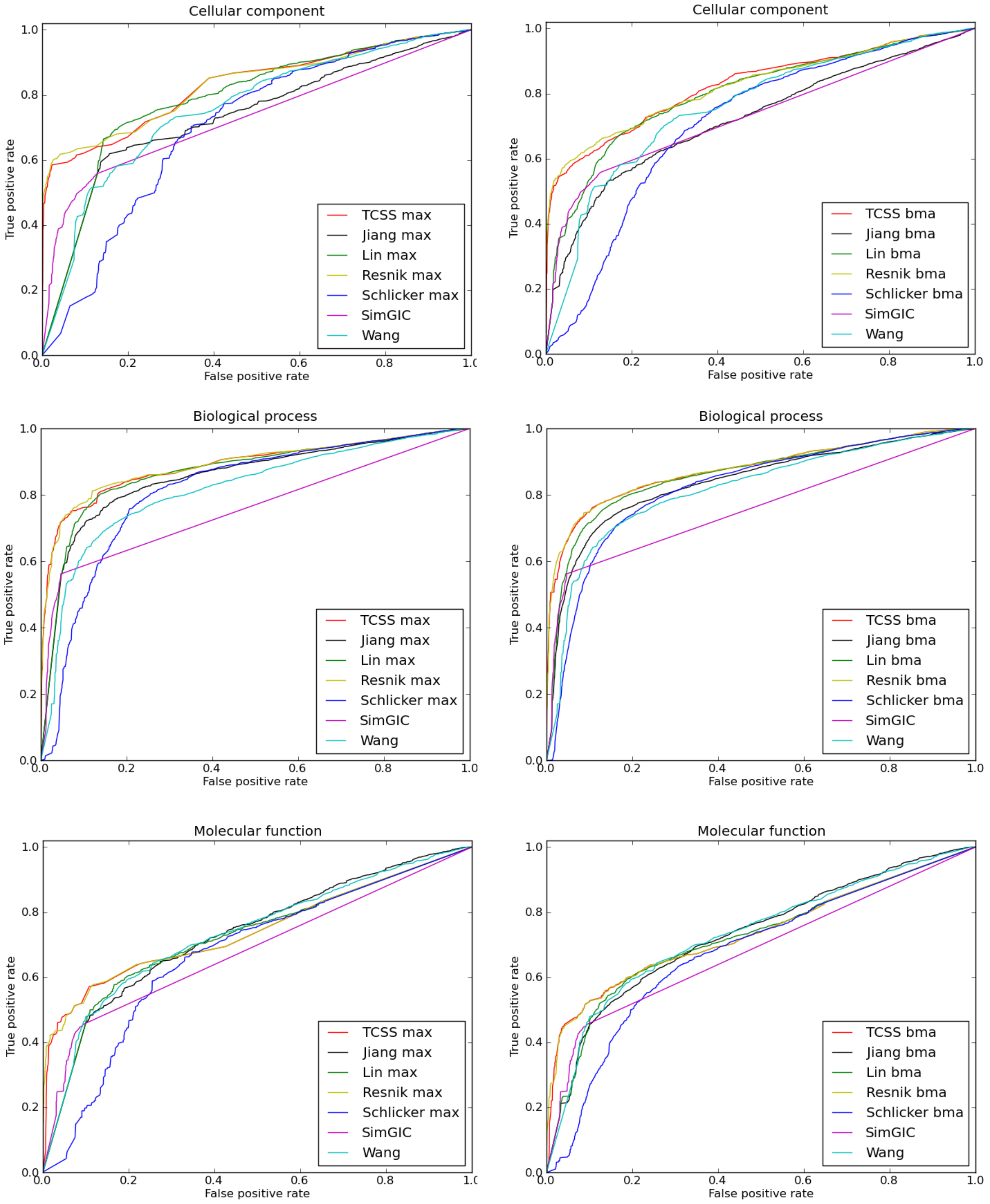

Supplementary figure S5 - F-score curves for *S. cerevisiae* PPI dataset (IEA+)

F_1 score (harmonic mean of precision and recall) evaluations of TCSS and Resnik semantic similarity measures at different cutoffs based on the *S. cerevisiae* PPI dataset derived from DIP are shown. The evaluation was performed using cellular component, biological process, molecular function ontology of GO. Best-match average (bma) and maximum (max) approaches for combining multiple annotations was used on dataset with (IEA+) electronic annotations. F_1 score reaches its best value at 1 and worst at 0. TCSS does better than Resnik for semantic similarity cutoff scores in all three ontologies.

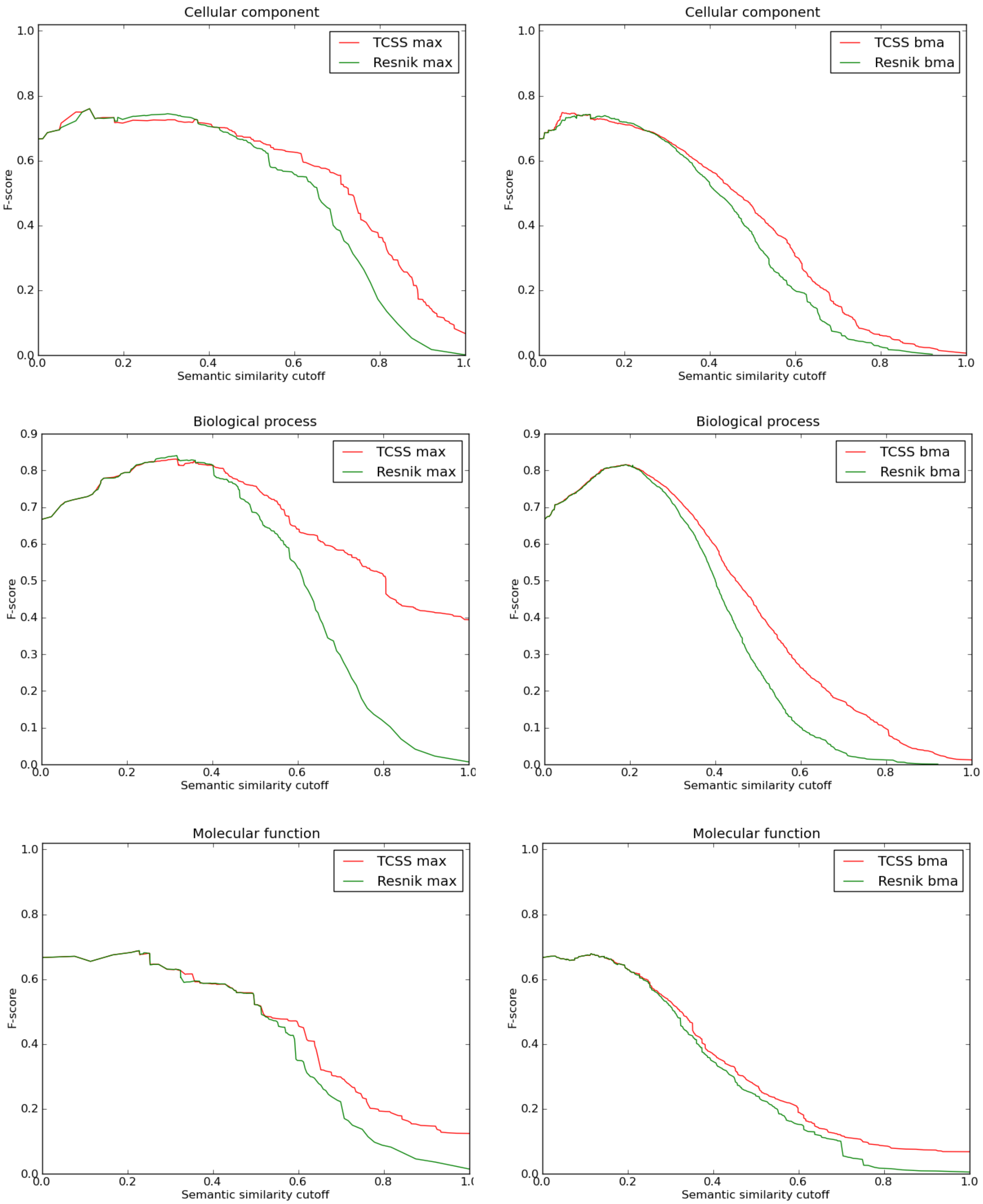

Supplementary figure S6 - ROC curves for *H. sapiens* PPI dataset (IEA-)

ROC evaluations of semantic similarity measures at different cutoffs based on the *H. sapiens* PPI dataset derived from DIP are shown. The evaluation was performed using cellular component, biological process, molecular function ontology of GO. Best-match average (bma) and maximum (max) approaches for combining multiple annotations were used on dataset without (IEA–) electronic annotations. TCSS & Resnik show best ROC profiles for all three ontologies.

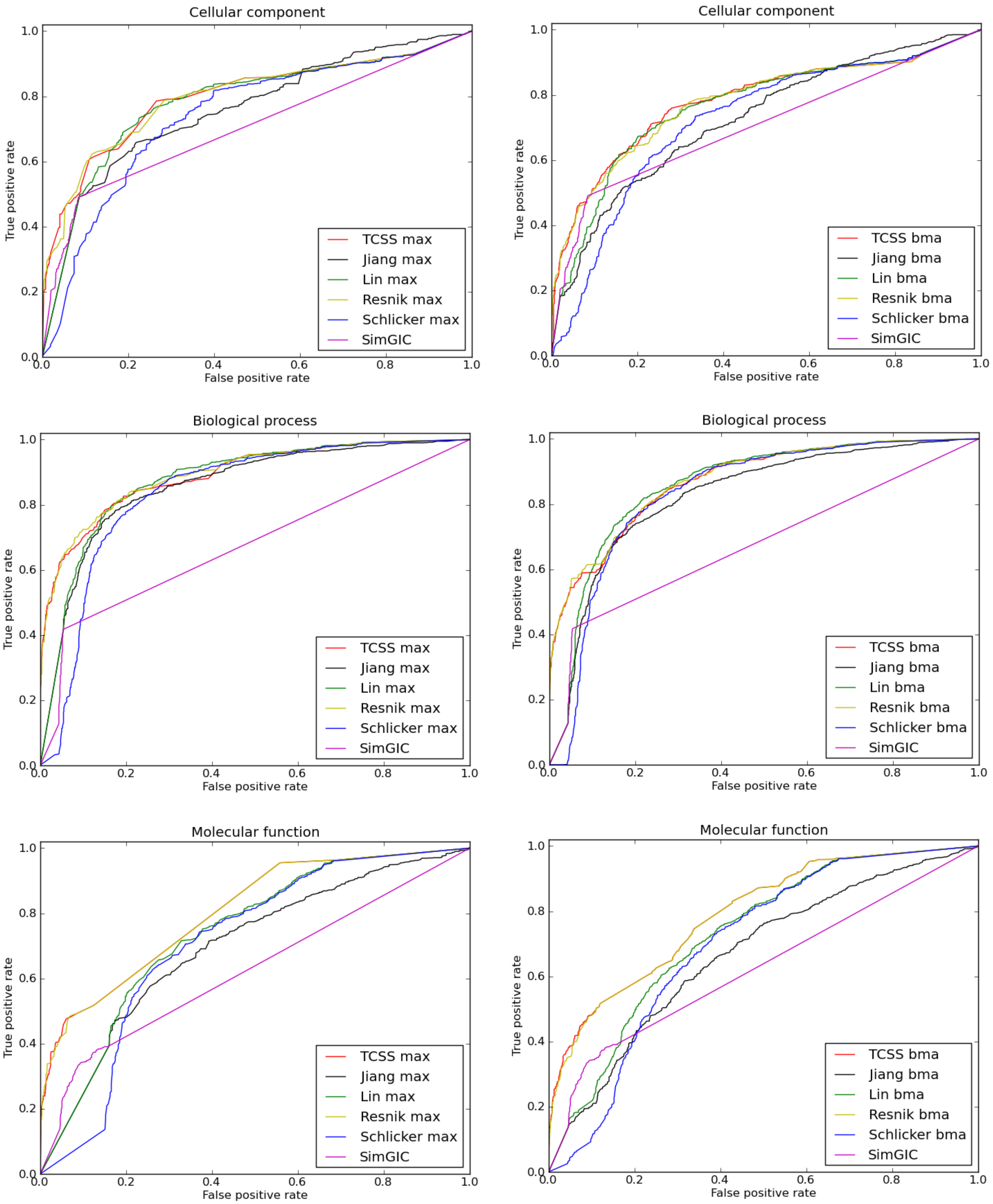

Supplementary figure S7 - F-score curves for *H. sapiens* PPI dataset (IEA-)

F_1 score (harmonic mean of precision and recall) evaluations of semantic similarity measures at different cutoffs based on the *H. sapiens* PPI dataset derived from DIP are shown. The evaluation was performed using cellular component, biological process, and molecular function ontologies of GO. Best-match average (bma) and maximum (max) approaches for combining multiple annotations were used on dataset without (IEA–) electronic annotations. F_1 score reaches its best value at 1 and worst at 0. TCSS does better than Resnik for semantic similarity cutoff scores in all three ontologies.

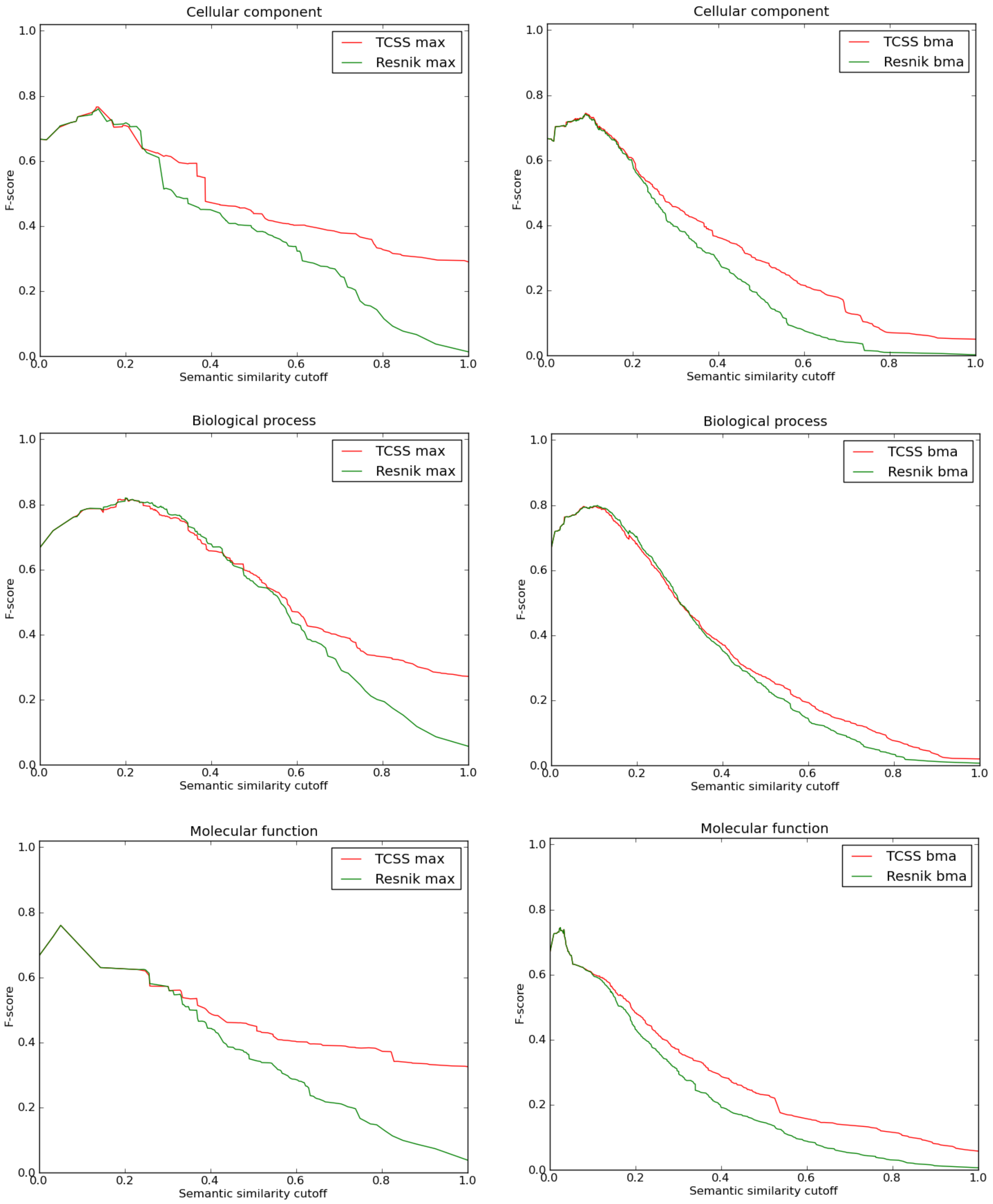

Supplementary figure S8 - ROC curves for *H. sapiens* PPI dataset (IEA+)

ROC evaluations of semantic similarity measures at different cutoffs based on the *H. sapiens* PPI dataset derived from DIP are shown. The evaluation was performed using cellular component, biological process, molecular function ontology of GO. Best-match average (bma) and maximum (max) approaches for combining multiple annotations were used on dataset with (IEA+) electronic annotations. TCSS & Resnik show best ROC profiles for all three ontologies.

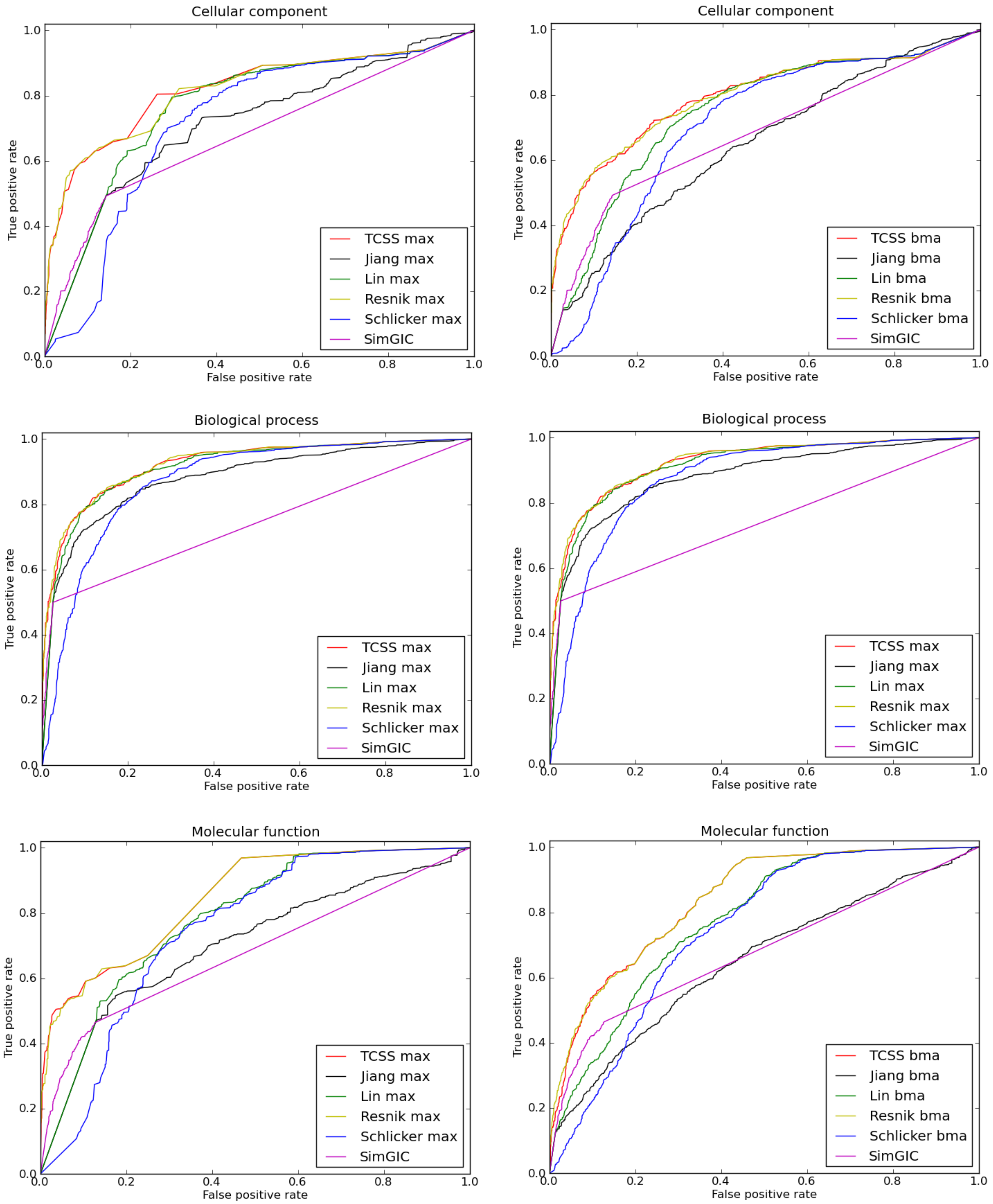

Supplementary figure S9 - F-score curves for *H. sapiens* PPI dataset (IEA+)

F_1 score (harmonic mean of precision and recall) evaluations of semantic similarity measures at different cutoffs based on the *H. sapiens* PPI dataset derived from DIP are shown. The evaluation was performed using cellular component, biological process, and molecular function ontologies of GO. Best-match average (bma) and maximum (max) approaches for combining multiple annotations were used on dataset with (IEA+) electronic annotations. F_1 score reaches its best value at 1 and worst at 0. TCSS does better than Resnik for semantic similarity cutoff scores in all three ontologies.

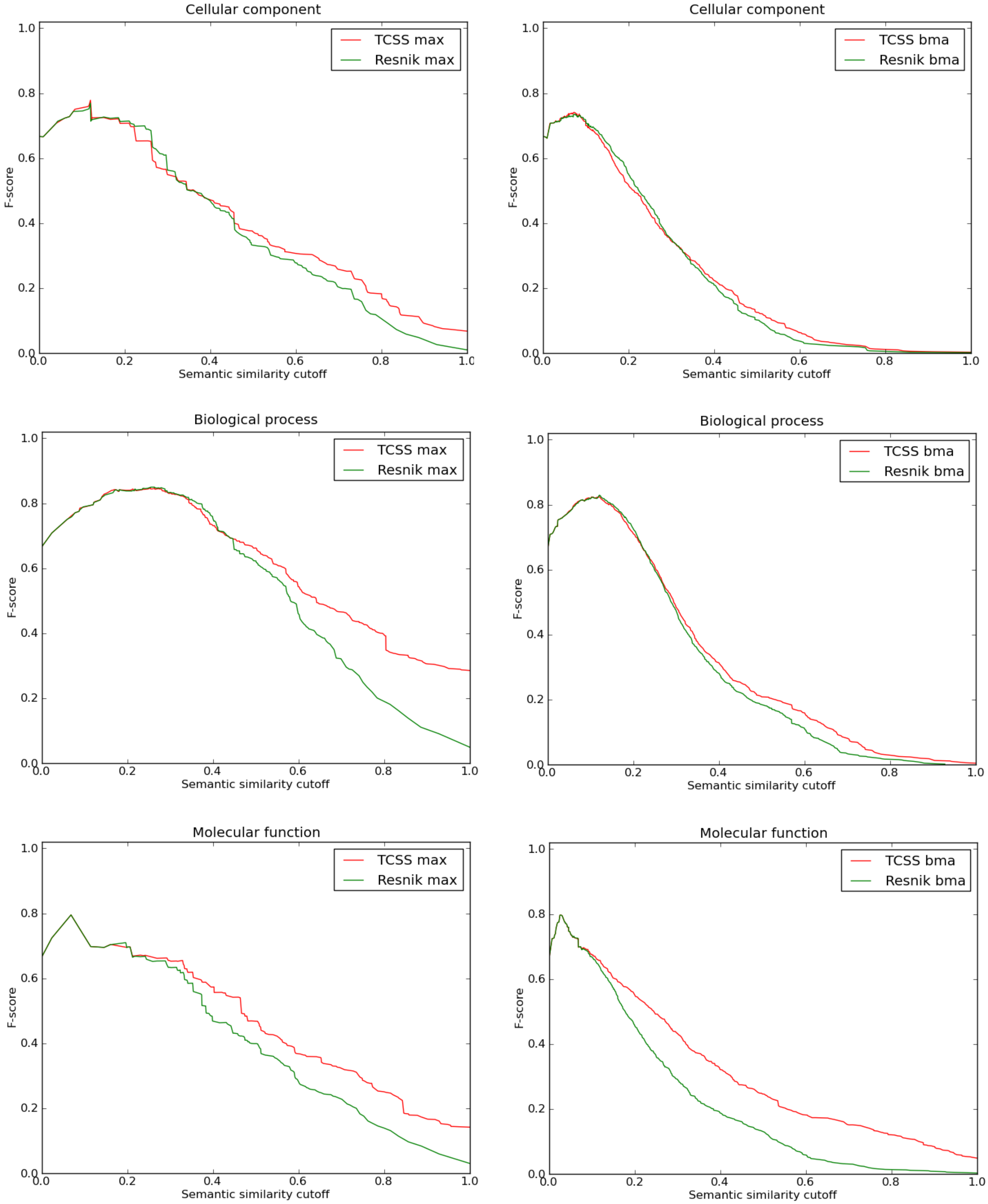

Supplementary figure S10 - Effect of topology cutoff on (ROC) AUC and F-score for *S. cerevisiae* PPI dataset (IEA-)

Change in AUC (TPR/FPR ROC) values and average F-scores with respect to topology cutoff under different settings. BMA stands for best-match average approach of combining multiple annotations and MAX stands for maximum approach. Test was conducted separately for cellular component (CC), biological process (BP), and molecular function (MF) ontologies without IEA (IEA-) annotations.

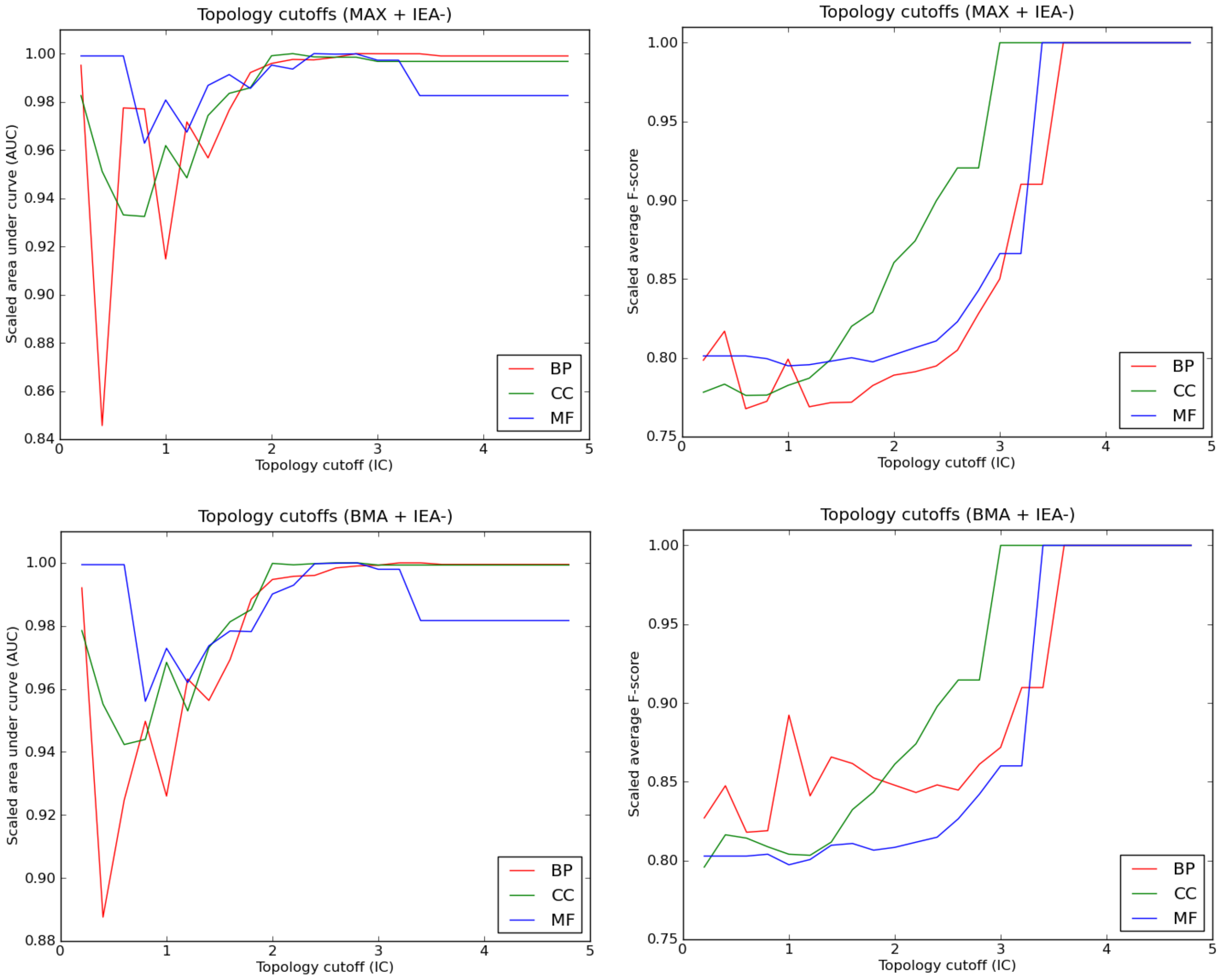

Supplementary figure S11 - Effect of topology cutoff on (ROC) AUC and F-score for *S. cerevisiae* PPI dataset (IEA+)

Change in AUC (TPR/FPR ROC) values and average F-scores with respect to topology cutoff under different settings. BMA stands for best-match average approach of combining multiple annotations and MAX stands for maximum approach. Test was conducted separately for cellular component (CC), biological process (BP), and molecular function (MF) ontologies with IEA (IEA+) annotations.

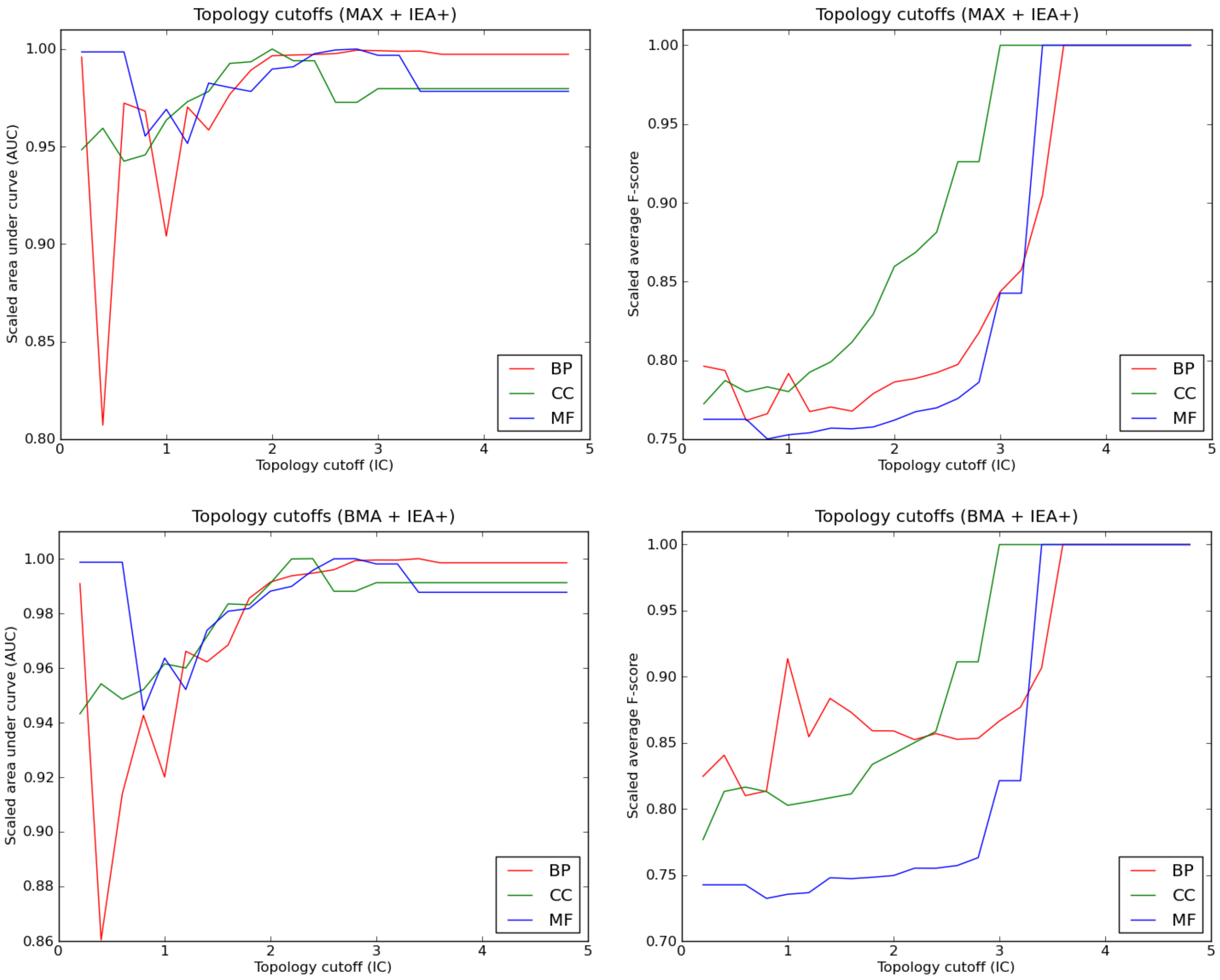

Supplementary figure S12 - Topology cutoff for *S. cerevisiae* PPI dataset

Topology cutoffs for cellular component (CC), biological process (BP), and molecular function (MF) ontologies were determined by evaluating AUC values and average F-scores at different cutoffs. The topology cutoff where both the AUC and average F-score maximized under different conditions is picked. Test was done with best-match average (bma) and maximum (max) approaches of combining multiple annotations on datasets with (IEA+) and without (IEA-) electronic annotations. Topology cutoff value chosen for CC is 2.4, BP is 3.6, and MF is 3.2 (marked by "×").

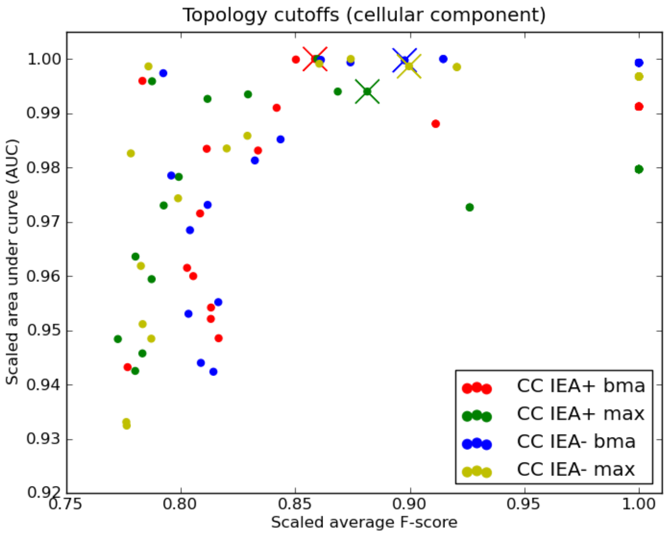

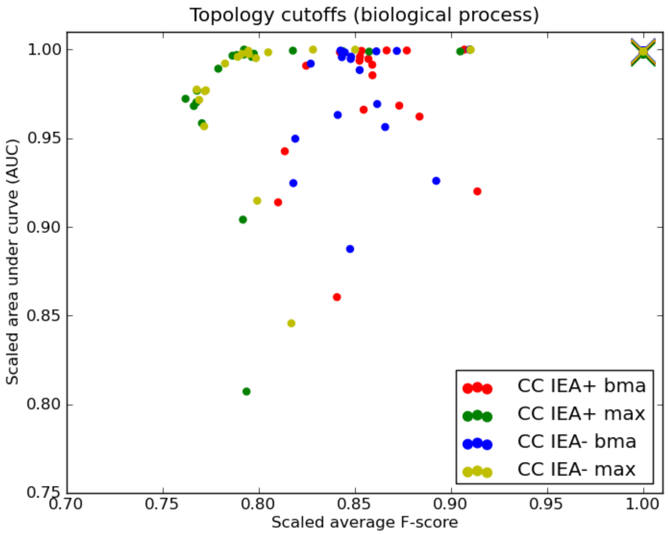

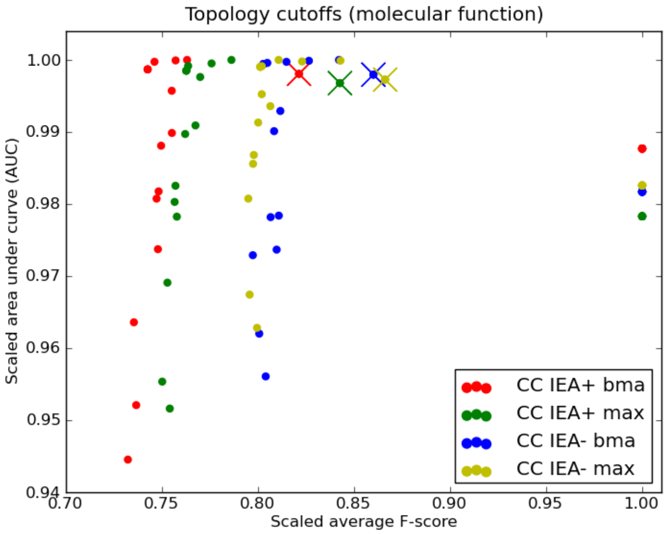

Supplementary figure S13 - Effect of topology cutoff on (ROC) AUC and F-score for *H. sapiens* PPI dataset (IEA-)

Change in AUC (TPR/FPR ROC) values and average F-scores with respect to topology cutoff under different settings. BMA stands for best-match average approach of combining multiple annotations and MAX stands for maximum approach. Test was conducted separately for cellular component (CC), biological process (BP), and molecular function (MF) ontologies without IEA (IEA-) annotations.

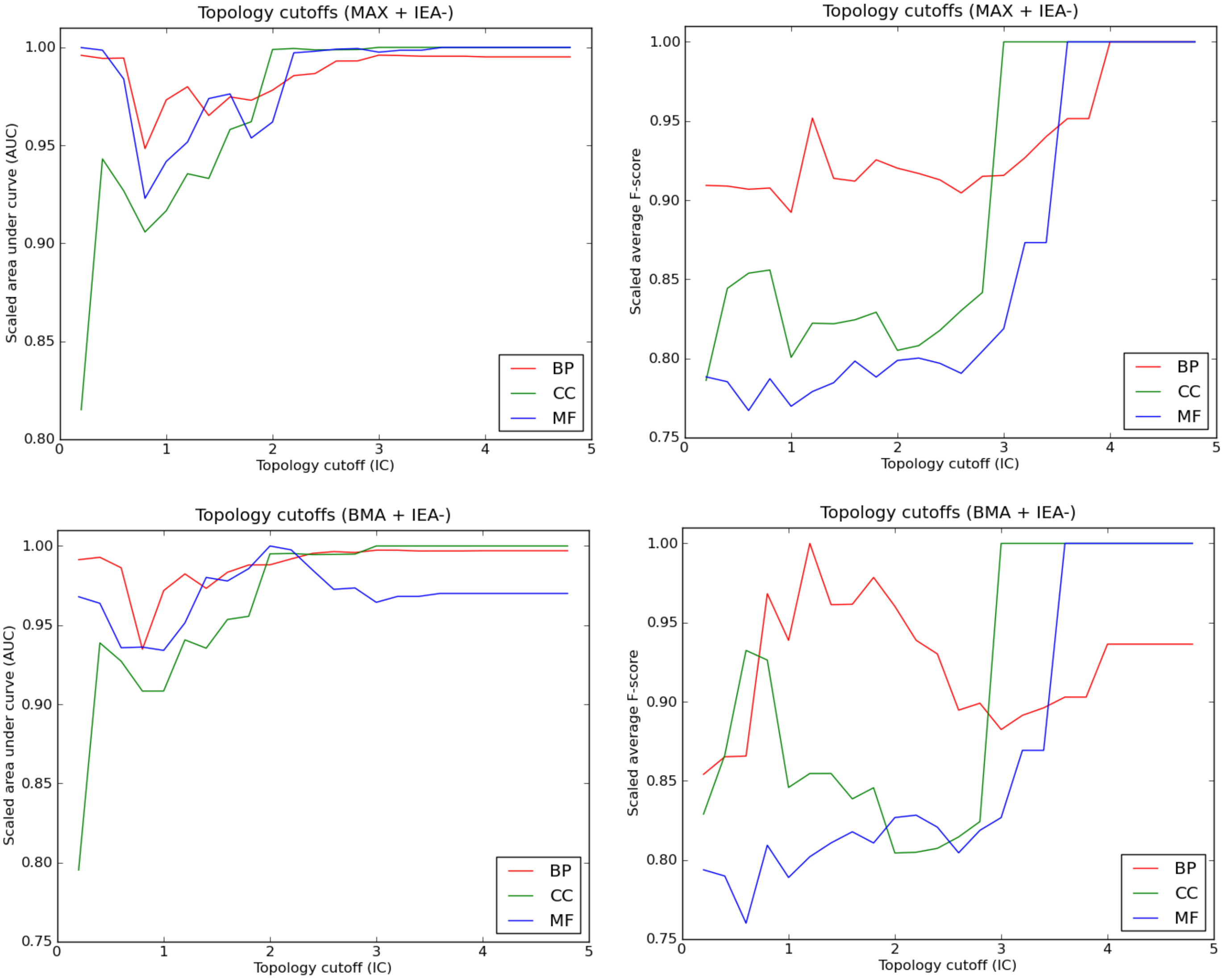

Supplementary figure S14 - Effect of topology cutoff on (ROC) AUC and F-score for *H. sapiens* PPI dataset (IEA+)

Change in AUC (TPR/FPR ROC) values and average F-scores with respect to topology cutoff under different settings. BMA stands for best-match average approach of combining multiple annotations and MAX stands for maximum approach. Test was conducted separately for cellular component (CC), biological process (BP), and molecular function (MF) ontologies with IEA (IEA+) annotations.

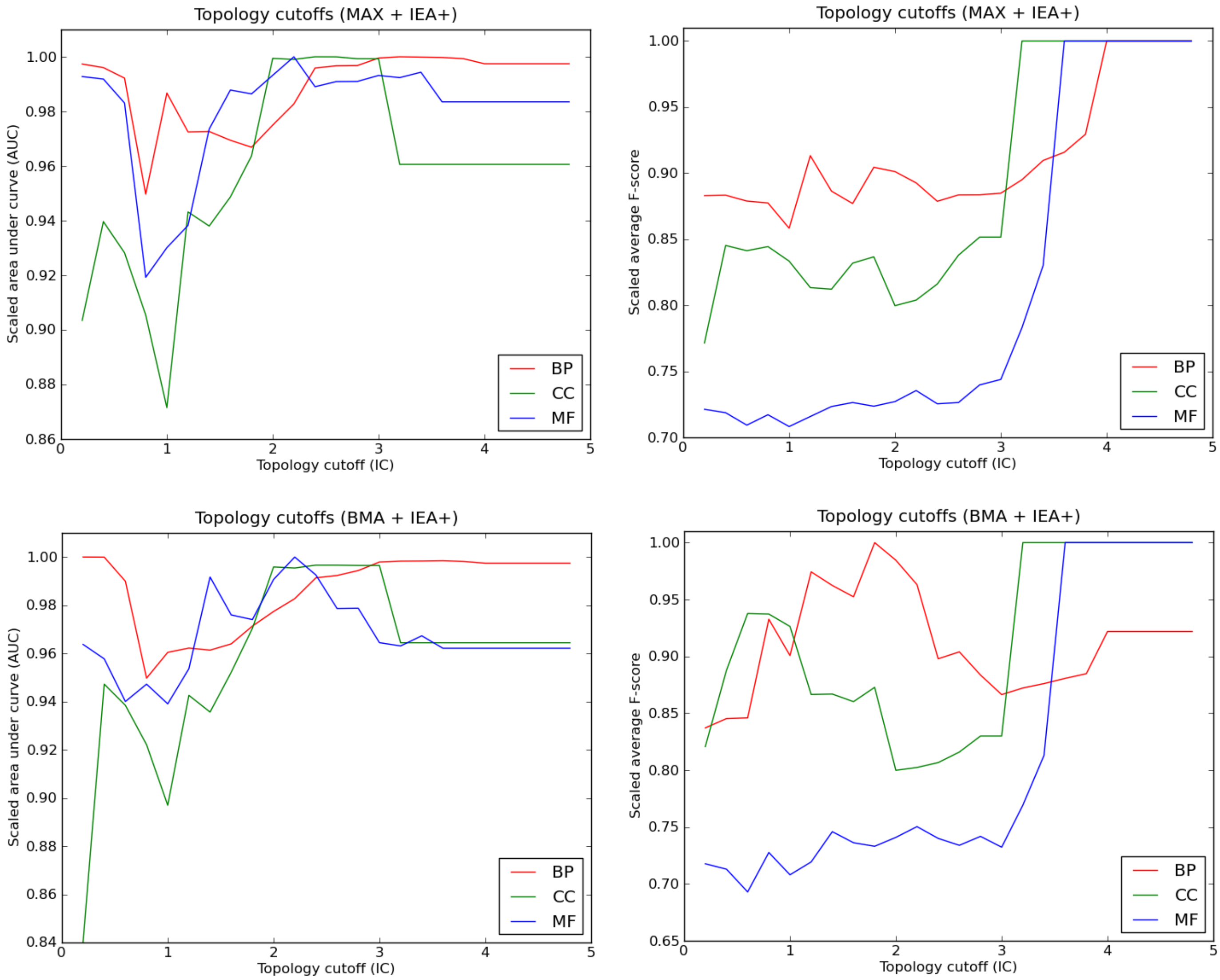

Supplementary figure S15 - Topology cutoff for *H. sapiens* PPI dataset

Topology cutoffs for cellular component (CC), biological process (BP), and molecular function (MF) ontologies were determined by evaluating AUC values and average F-scores at different cutoffs. The topology cutoff where both the AUC and average F-score maximized under different conditions is picked. Test was done with best-match average (bma) and maximum (max) approaches of combining multiple annotations on datasets with (IEA+) and without (IEA-) electronic annotations. Topology cutoff value chosen for CC is 3.0, BP is 4.0, and MF is 3.6 (marked by "×").

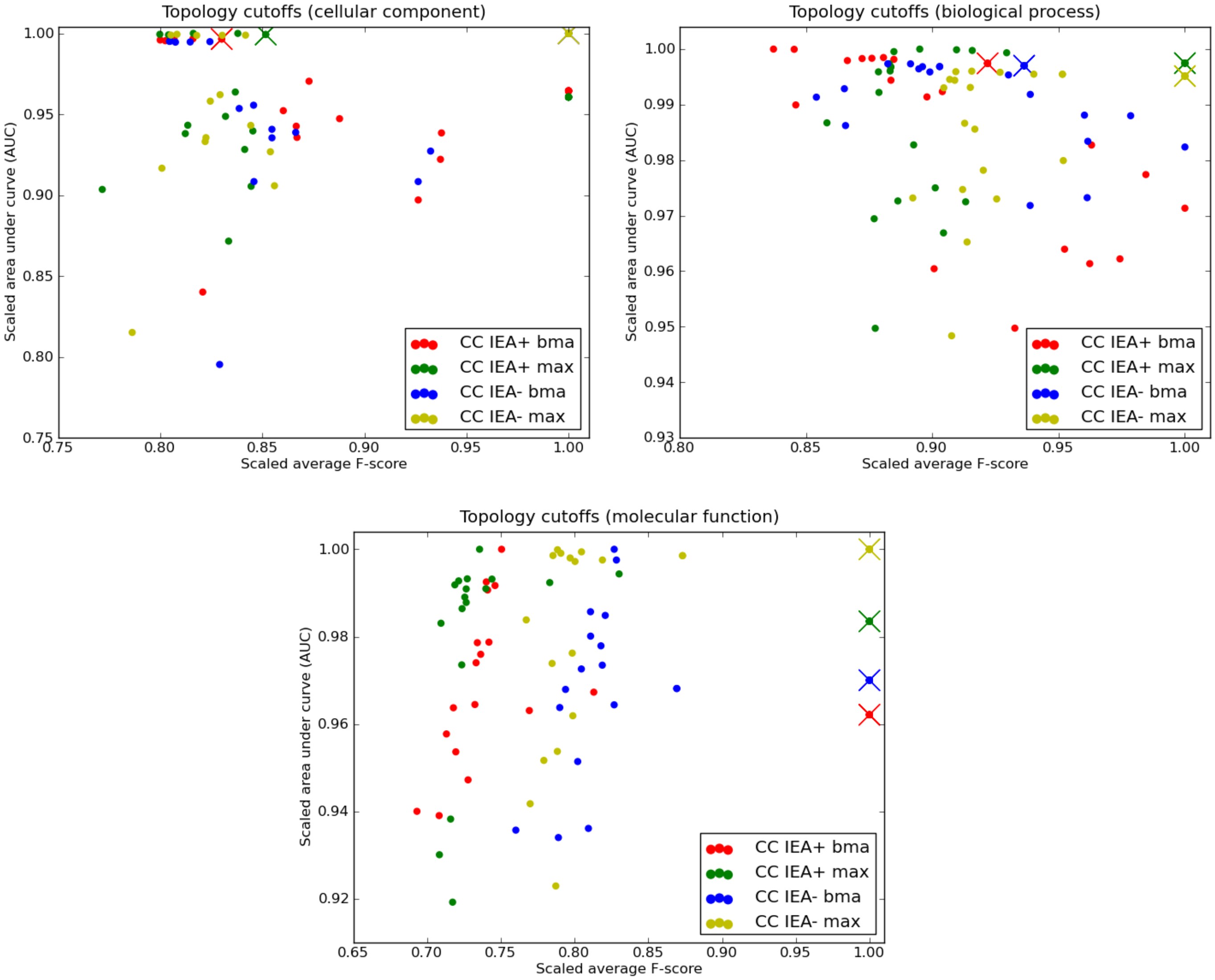

Supplementary figure S16 - Correlation with gene expression

Pearson correlation between gene expression similarity and semantic similarity on *S. cerevisiae* dataset are shown. The evaluation was performed for cellular component, biological process, and molecular function ontologies of GO. Best-match average (bma) approach for combining multiple GO annotations was used. TCSS showed best correlation with gene expression in all three ontologies.

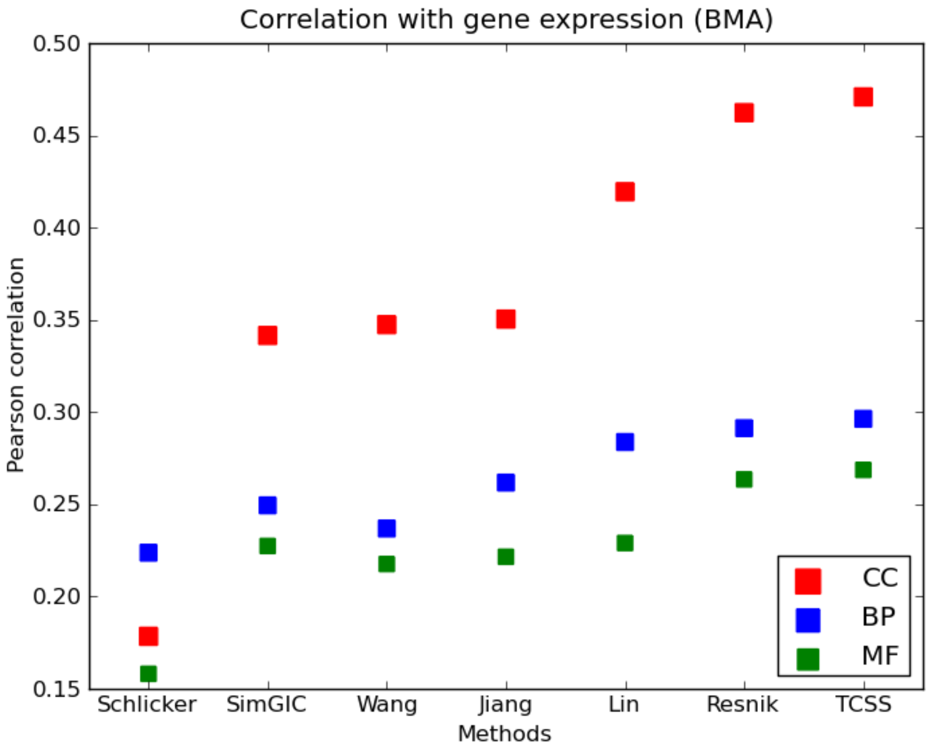

Supplementary figure S17 - Correlation with CESSM dataset

Correlation between semantic similarity and sequence, enzyme commission (EC), protein family (Pfam) similarity using online CESSM tool. The evaluation was performed for cellular component (CC), biological process (BP), and molecular function ontologies (MF) of GO. Best-match average (bma) approach for combining multiple GO annotations was used on the dataset without (IEA–) electronic annotations. TCSS showed best correlation with EC & Pfam similarity for CC ontology and same as Resnik's for MF and BP ontologies.

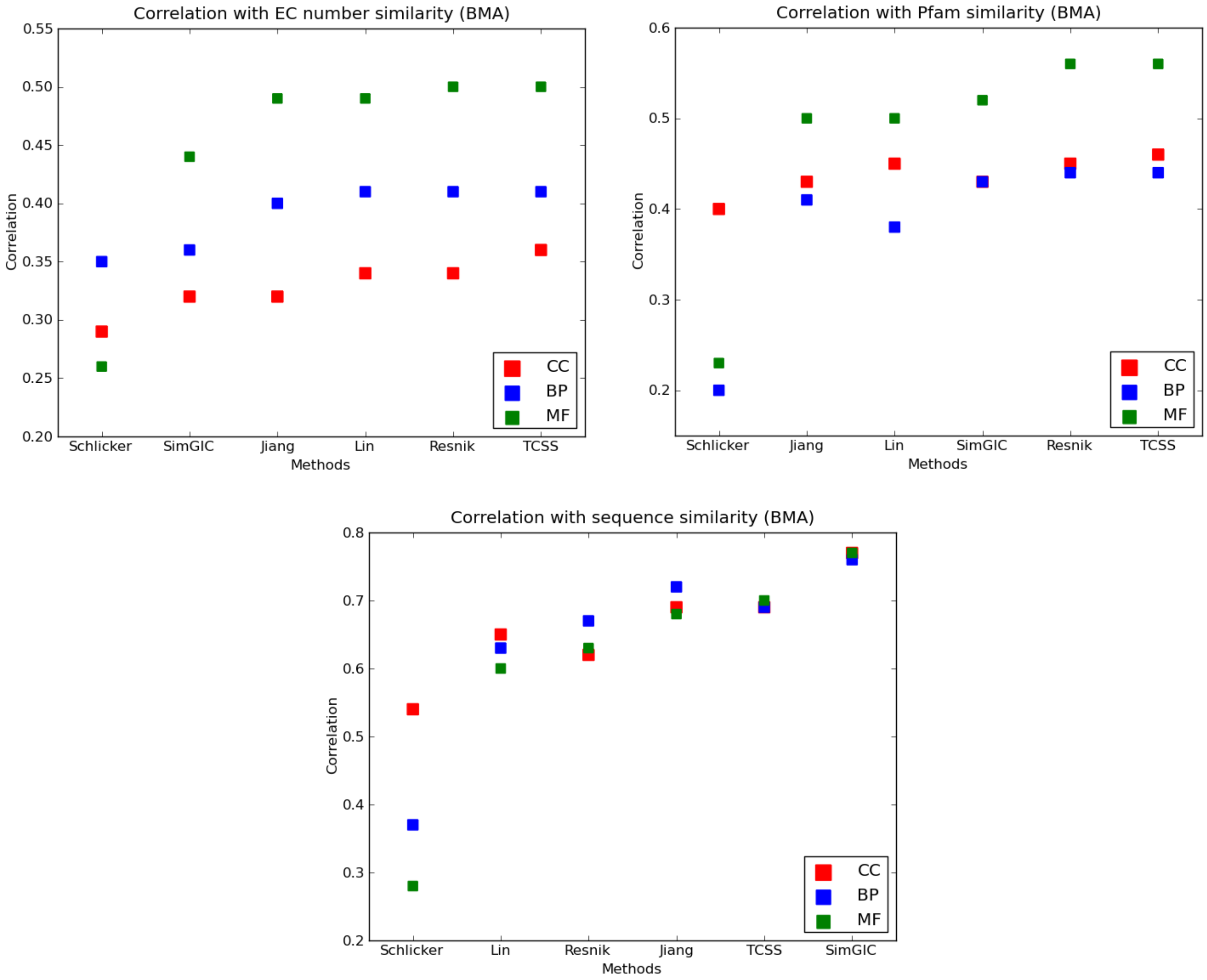

Supplementary table S1 - Area under ROC curves for *H. sapiens* PPI dataset

Area under ROC curves for *H. sapiens* PPI dataset. The tests were performed separately for cellular component (CC), biological process (BP), and molecular function (MF) ontologies. *Best-match average* and *maximum* approaches were used for datasets "with (IEA+) and without (IEA–)" electronic annotations.

		IEA–			IEA+		
		CC	BP	MF	CC	BP	MF
TCSS	max	**0.80**	**0.89**	**0.80**	**0.82**	**0.92**	**0.85**
	bma	0.78	0.87	0.79	0.79	0.90	0.84
Resnik	max	**0.80**	**0.89**	**0.80**	0.81	**0.92**	0.84
	bma	0.77	0.87	0.79	0.79	0.90	0.84
Lin	max	0.78	0.88	0.74	0.76	0.91	0.78
	bma	0.76	0.86	0.73	0.75	0.88	0.78
Jiang	max	0.76	0.86	0.70	0.71	0.88	0.70
	bma	0.73	0.83	0.67	0.65	0.80	0.65
Schlicker	max	0.74	0.84	0.71	0.72	0.87	0.75
	bma	0.72	0.83	0.70	0.70	0.85	0.74
SimGIC		0.70	0.68	0.63	0.68	0.74	0.68

Supplementary table S2 - Gene expression datasets from GeneMANIA

Gene Expression Omnibus (GEO) identifiers, series title, pubmed ids of gene expression datasets downloaded from GeneMANIA.

GEO id	Series title	Pubmed id
GSE1311	YDRseries1, Yeast desiccation / rehydration time course	16332871
GSE1312	YDRseries2, Yeast desiccation / rehydration time course	16332871
GSE1313	YDRseries3, Yeast desiccation / rehydration time course	16332871
GSE1639	Rpd3 and histone H3 and H4 deletions/mutations	15456858
GSE1693	A novel response to microtubule perturbation in meiosis	15899877
GSE1723	Two-dimensional transcriptome analysis in chemostat cultures of S. cerevisiae	15496405, 17241460, 12414795
GSE1814	Transcriptional effects of the TOR2-controlled signaling function	15476558, 16959779
GSE1938	Phosphomannose isomerase gene (PMI40) deletion strain cultivated in varying initial mannose concentrations	15520001
GSE1975	Simultaneous genotyping, gene expression measurement, and detection of allele-specific expression	15687292
GSE2076	leu3p dependent transcription	15949974
GSE2224	Experimental condition	15878181
GSE2343	TFIIH mutants treated with methyl methanesulfonate	15837426
GSE3076	Impact of Nonsense-mediated mRNA Decay on the Global Expression Profile of Budding Yeast	17166056
GSE3431	Logic of the yeast metabolic cycle	16254148
GSE3806	Histone H2B^3-32, H2B K->G, H2B^3-37, and H2B^30-37 mutations	16648479
GSE3821	Short term perturbation	16969341
GSE4135	Wild type yeast and H3del(1-28) and H4del(2-26) yeast grown in complete synthetic media	16461773
GSE4669	Response of yeast to saponin treatment	16870766
GSE5238	SFP1 dependent transcription	18174152
GSE5301	Expression data from yeast treated with enediynes compared to gamma radiation	17163986
GSE6073	Rap1 and Abf1 DNA-binding ts mutants and wild type after 1 hr at 37 C	17158163
GSE6190	Temperature-dependent transcriptional response under anaerobic C and N limitations in Yeast	17928405
GSE6405	Transcriptional responses of yeast to preferred and non-preferred nitrogen sources in C-lim chemostat cultures	17419774
GSE7660	Sch9 Is a Major Target of TORC1 in Saccharomyces cerevisiae	17560372
GSE7820	Transcript and Proteomic Analyses of Wild-Type and GPA2 Mutant Saccharomyces cerevisiae Strains	17700863
GSE8187	Adaptation of S. cerevisiae to fermentative conditions	18304306
GSE8536	The response of Saccharomyces cerevisiae to stress throughout a 15-day wine fermentation	18215224
GSE8761	Transcriptional profiling of ribosomal protein knockouts	17981122
GSE8825	Coordination of Growth Rate, Cell Cycle, Stress Response and Metabolic Activity in Yeast	17959824
GSE8895	Role of Transcriptional Regulation in Controlling Fluxes in Central Carbon Metabolism of Saccharomyces cerevisiae	14630934
GSE8900	Genome-wide transcriptional responses of Saccharomyces cerevisiae to high carbon dioxide concentrations	15780657
GSE9217	Transcriptomes for different level of glucose	18679056
GSE9302	A perturbation in the system leads to period doubling	17043222
GSE9423	The Oxidative Stress Response of a Lager Brewing Yeast Strain during Industrial Propagation and Fermentation	18373683
GSE9482	GAL-NMD2	18087042
GSE9590	Saccharomyces cerevisiae TPP 2-oxo acid decarboxylases	18281432
GSE9644	Glucose Pulse to sfp1delta continuous cultures	18524923
GSE11452	Saccharomyces cerevisiae chemostat steady state microarray compendium	19173729
GSE12890	Xylose metabolism in recombinant Saccharomyces cerevisiae	18533012
